# FOXO3 targets are reprogrammed as Huntington's disease neural cells and striatal neurons face senescence with p16^INK4a^ increase

**DOI:** 10.1111/acel.13226

**Published:** 2020-11-06

**Authors:** Jessica Voisin, Francesca Farina, Swati Naphade, Morgane Fontaine, Kizito‐Tshitoko Tshilenge, Carlos Galicia Aguirre, Alejandro Lopez‐Ramirez, Julia Dancourt, Aurélie Ginisty, Satish Sasidharan Nair, Kuruwitage Lakshika Madushani, Ningzhe Zhang, François‐Xavier Lejeune, Marc Verny, Judith Campisi, Lisa M. Ellerby, Christian Neri

**Affiliations:** ^1^ Centre National de la Recherche Scientifique UMR 8256 Institut National de la Santé et de la Recherche Médicale ERL U1164 Assistance Publique‐Hôpitaux de Paris Brain‐C Lab Sorbonne Université Paris France; ^2^ Buck Institute for Research on Aging Novato CA USA; ^3^ Lawrence Berkeley National Laboratory Berkeley CA USA

**Keywords:** neurodegenerative disease, neuronal differentiation, neuronal senescence, response mechanisms, temporal dynamics

## Abstract

Neurodegenerative diseases (ND) have been linked to the critical process in aging—cellular senescence. However, the temporal dynamics of cellular senescence in ND conditions is unresolved. Here, we show senescence features develop in human Huntington's disease (HD) neural stem cells (NSCs) and medium spiny neurons (MSNs), including the increase of p16^INK4a^, a key inducer of cellular senescence. We found that HD NSCs reprogram the transcriptional targets of FOXO3, a major cell survival factor able to repress cell senescence, antagonizing *p16^INK4a^* expression *via* the FOXO3 repression of the transcriptional modulator ETS2. Additionally, *p16^INK4a^* promotes cellular senescence features in human HD NSCs and MSNs. These findings suggest that cellular senescence may develop during neuronal differentiation in HD and that the FOXO3‐ETS2‐p16^INK4a^ axis may be part of molecular responses aimed at mitigating this phenomenon. Our studies identify neuronal differentiation with accelerated aging of neural progenitors and neurons as an alteration that could be linked to NDs.

## Graphical abstract

Increasingly pronounced senescence features develop in human Huntington's disease (HD) neural stem cells and striatal neurons, including the increase of p16^INK4a^. The FOXO3/ß‐catenin complex can oppose these cellular senescence features via transcriptional reprogramming in a Ryk‐dependent manner, antagonizing *p16^INK4a^* expression via the repression of the transcriptional modulator ETS2. The HD brain neural lineage may thus face a continuous senescence process.

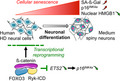

## 1 INTRODUCTION

FOXO (Forkhead Box O) transcription factors are key regulators of longevity that engage several repair mechanisms to promote the survival of cells facing stress (Martins, Lithgow, & Link, 2016; Salih & Brunet, 2008). In response to overwhelming stress, FOXO factors may trigger cell death, for example, target senescent cells to apoptosis via interaction with the p53 protein (Baar et al., 2017). In neurodegenerative diseases (ND), FOXO factors such as DAF‐16 and FOXO3 may protect against the cytotoxicity of Huntingtin (HTT) (Parker et al., 2012; Tourette et al., 2014), SOD1 and p150^Glued^ (Mojsilovic‐Petrovic et al., 2009), α‐synuclein (Pino et al., 2014), and Aß (Cohen et al., 2009). Interestingly, NDs have been linked to cellular senescence, particularly that of glial cells (Bussian et al., 2018; Chinta et al., 2018; Musi et al., 2018; Zhang et al., 2019). However, the temporal dynamics of cellular senescence in NDs and the role of FOXO gene regulation in this context are unresolved, limiting our capacity to target the detrimental effects of cellular senescence in NDs.

We hypothesized that FOXO gene regulation might be able to oppose cellular senescence in ND conditions. We tested this hypothesis in human cell models of Huntington's disease (HD), a genetic yet a primarily late‐onset ND caused by CAG expansion in *HTT*. We focused on FOXO3, a FOXO factor that is neuroprotective in HD (Tourette et al., 2014). Although FOXO3 is pivotal to neuronal homeostasis in HD, human FOXO3 targets are unknown, including in ND conditions. Here, we found that human HD induced pluripotent stem cell (iPSC)‐derived neural stem cells (NSC) reprogram FOXO3 targets in the context of cellular senescence features that are acquired at the time of neuronal differentiation and that are more pronounced in medium spiny neurons (MSNs). These features include the increase of p16^INK4a^, a key inducer of cellular senescence (Baker et al., 2016). Remarkably, FOXO3 target reprogramming represses the transcription modulator and *p16^INK4a^* activator ETS2 (Irelan et al., 2009), which antagonizes *p16^INK4a^* expression and which may represent an adaptive response as *p16^INK4a^* promotes the senescence of human HD NSCs and MSNs. Together, these data reveal that cellular senescence may develop during neuronal differentiation in HD, affecting striatal neurons, and that FOXO gene regulation may tip the balance away from the detrimental consequences of cellular senescence via *ETS2*‐*p16^INK4a^*, providing a rationale and strategy for targeting cellular senescence during the early phases of NDs, before the onset of overt neuronal injuries and cell death, a crucial need in HD and other NDs.

## 2 RESULTS

### 2.1 Ryk‐ICD binds to Armadillo repeats 9‐10 of ß‐catenin

**FIGURE 1:**
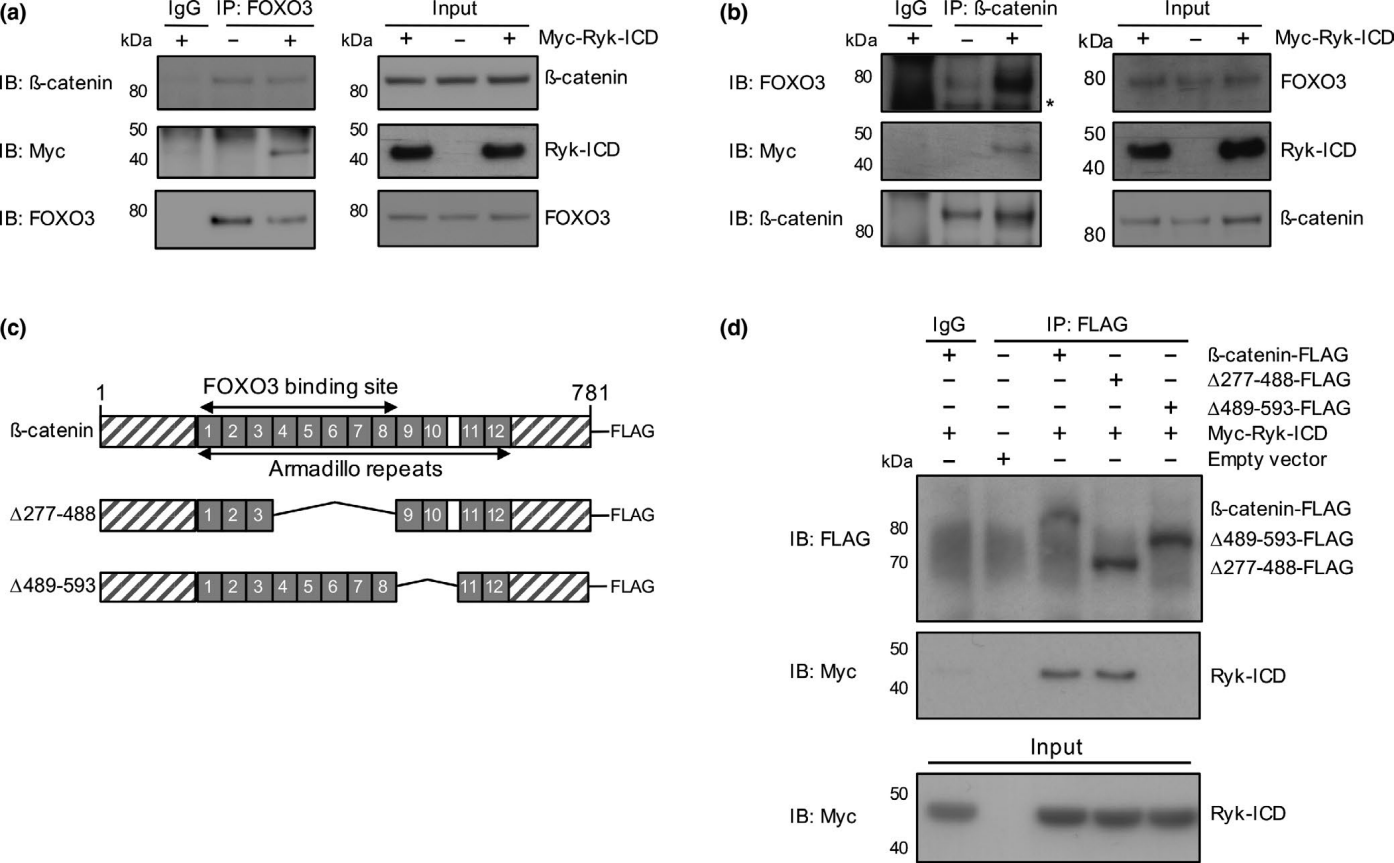
FOXO3, ß‐catenin, and Ryk‐ICD form a protein complex in HEK293T cells. The antibodies used for immunoprecipitation (IP) and for immunoblotting (IB) are indicated across panels. (a) FOXO3, ß‐catenin, and Ryk‐ICD co‐precipitate in pull‐down experiments. For negative control, an IgG isotype was used. Representative Western blots for IP of endogenous FOXO3. (b) FOXO3, ß‐catenin and Ryk‐ICD co‐precipitate in pull‐down experiments. For negative control, an IgG isotype was used. Representative Western blots for IP of endogenous ß‐catenin. (c) Deletion mapping of the Armadillo repeat region in ß‐catenin. Ryk‐ICD binds to Armadillo repeats 9‐10 of ß‐catenin. HEK293T cells were transfected with Myc‐Ryk‐ICD construct and the indicated deletion mutants (∆277‐488, ∆489‐593). For negative control, an IgG isotype was used. (d) Representative Western blots for IP of wild‐type and mutant ß‐catenin‐FLAG constructs shown in (c)

In HD, FOXO3 neuroprotection is altered by increased mRNA and protein expression of Ryk (Tourette et al., 2014), a Wnt receptor important for axon guidance and neurogenesis (Andre et al., 2012). This effect, a consequence of gene deregulation in HD, is mediated by the Ryk intracellular domain (Ryk‐ICD) in the nucleus where Ryk‐ICD binds to the FOXO3 partner ß‐catenin (Tourette et al., 2014). To determine how Ryk‐ICD alters FOXO3‐ß‐catenin homeostasis, we performed co‐immunoprecipitation assays in HEK293 T cells. We overexpressed a Myc‐tagged Ryk‐ICD fragment, as these cells normally produce relatively small amounts of this gamma‐secretase cleavage product (Tourette et al., 2014). The Myc‐tagged Ryk‐ICD fragment co‐precipitated with ß‐catenin when endogenous FOXO3 was targeted by the immunoprecipitating antibody (Figure 1a) as well as with FOXO3 when endogenous ß‐catenin was targeted by the immunoprecipitating antibody (Figure 1b). Thus, Ryk‐ICD may be an integral part of the ß‐catenin/FOXO3 complex.

We mapped Ryk‐ICD binding site to ß‐catenin. The results suggest the Ryk‐ICD binding site encompasses Armadillo repeats 9‐10 on ß‐catenin (Figure 1c). The FOXO3 binding site was previously mapped to ß‐catenin Armadillo repeats 1‐8 (Essers et al., 2005; Hoogeboom et al., 2008) (Figure 1c,d), suggesting that Ryk‐ICD binds ß‐catenin adjacent to FOXO3 to form a tripartite protein complex.

### 2.2 Human HD NSCs reprogram FOXO3 targets

Having shown that Ryk signaling may modulate FOXO3 gene regulation through spatial/allosteric modifications of the ß‐catenin/FOXO3 complex, we used massively paralleled RNA sequencing (RNA‐seq) and chromatin immunoprecipitation followed by sequencing (ChIP‐seq) to identify FOXO3 direct targets (F3Ts) in human HD cells. A human‐induced pluripotent stem cell (iPSC) model of HD, in which isogenic cells express mutant (72Q/19Q) *HTT* (HD) or CAG‐corrected (21Q/19Q) *HTT* (C116) was used (An et al., 2012; Ring et al., 2015). HD and C116 NSCs were treated with Ryk or scrambled sequence siRNAs. As expected, HD NSCs showed increased (1.2 fold) Ryk mRNA levels (Figure S1A) and a 2 fold in human HD MSNs (Figure 6b) whereas ß‐catenin and *FOXO3* mRNA levels are similar in HD and C116 cells (Figure S1A, middle and right panels). We then induced FOXO3 nuclear translocation (see Section 4) in Ryk siRNA‐silenced NSCs (Figure S1B,D) prior to collecting RNA‐seq and FOXO3 ChIP‐seq data.

Much of FOXO3 transcriptional activity can be due to binding enhancers (Eijkelenboom, Mokry, de Wit, et al., 2013), and there is a significant association between gene regulation and FOXO3 binding up to 20 kb from transcriptional start sites in human cells (Eijkelenboom, Mokry, Smits, Nieuwenhuis, & Burgering, 2013). We thus defined FOXO3 direct targets as genes that (a) show FOXO3 binding at promoter and enhancer regions (±20 kb) as determined by ChIP‐seq data and (b) are up‐ or down‐regulated upon FOXO3 induction into the nucleus as determined by RNA‐seq data (Table S1/sheet 1). Additionally, we used RNA‐seq data upon FOXO3 knockdown (Table S1/sheet 2). However, ß‐catenin transcriptional activity may bypass the absence of TCF/LEF (Doumpas et al., 2019) and, possibly, that of FOXO3 (Essers et al., 2005). Hence, FOXO3 nuclear induction and knockdown could differently alter gene regulation, which calls for caution in using FOXO3 knockdown data to prioritize F3Ts. We thus used these data as a *bona fide* criterion for defining two classes of F3Ts, that is, those identified by (a) FOXO3 nuclear induction (F3T‐IN) and (b) FOXO3 nuclear induction and FOXO3 knockdown (F3T‐IN‐KD) (Table S1).

**FIGURE 2:**
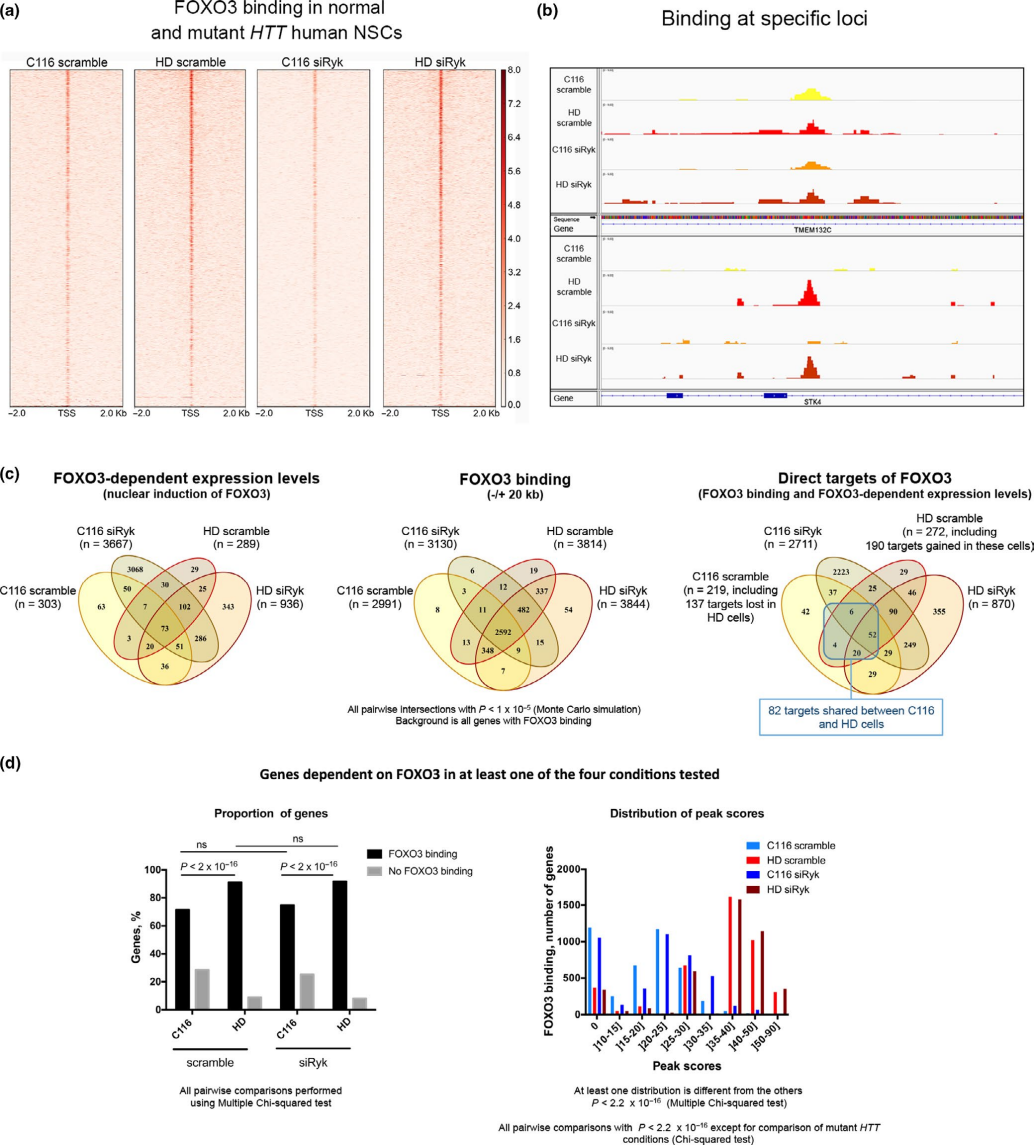
FOXO3 binding and gene regulation in human NSCs expressing normal or mutant *HTT* with or without Ryk silencing. (a) Enrichment of FOXO3 binding around the transcriptional start sites (TSSs) (±2 kb) in human NSCs expressing normal (C116: 19Q/21Q) or mutant *HTT* (HD: 72Q/21Q) and treated with Ryk siRNA‐1 (siRyk) or scrambled RNA (scramble). The color scale is chip signal intensity with maximum set as 8.0. (b) FOXO3 binding at specific loci in human C116 or HD NSCs. The upper panel is a FOXO3 binding site present in C116 and HD cells at the *TMEM132C* locus. The lower panel is a FOXO3 binding site gained in HD cells at the *STK4* locus. (c) Venn diagrams depicting F3 gene regulation across the 4 conditions tested. The left panel shows FOXO3‐dependent genes (RNA‐seq data). The middle panel shows FOXO3 binding (ChIP‐seq data). The right panel shows the distribution of F3Ts, highlighting an increase in the number of F3Ts upon Ryk silencing in C116 (*p* < 2.2e‐16) and HD (*p* < 2.2e‐16) cells. (d) FOXO3 binding for the FOXO3‐dependent genes. The left panel shows the percentages of genes with binding or no binding. Multiple chi‐square tests were performed using the R function pairwise.prop.test. The right panel shows the signal (peak score) distributions. Chi‐squared test was performed for global and pairwise comparisons of the distributions with the R function chisq.test

The F3T‐IN data indicated that, of the 219 F3Ts in C116 NSCs, 137 were lost in HD NSCs and that, among 272 F3T‐INs in HD cells, 190 were not present in C116 cells (Figure 2a,c, Figure S2). The gain of F3Ts in HD cells was accompanied by an increase in the proportion of genes with FOXO3 binding (±20 kb) (Figure 2d, left panel) and in FOXO3 binding levels (Figure 2d, right panel), indicating that FOXO3 occupancy is elevated in HD cells. Silencing Ryk greatly increased the number of F3T‐INs in C116 cells and to a lower extent in HD cells, unrelated to changes of FOXO3 binding (Figure 2c,d). Silencing Ryk also resulted in some loss of F3T‐INs in C116 cells, regardless of the type of FOXO3 regulation (Table S2/sheet‐1). Increased FOXO3 occupancy was also true for F3T‐INs that are gained (Table S2/Sheet‐2) or conserved (Table S2/Sheet‐3) in HD NSCs. Thus, Ryk signaling may function as a co‐repressor or co‐activator of FOXO3, with distinct effects on F3Ts between C116 and HD genotypes. In HD cells, 111 F3Ts are dependent on Ryk (Figure 2c, right panel). Together, these results suggest that F3Ts are reprogrammed in response to HD during neurogenesis and this response cannot be fully attributed to higher FOXO3 occupancy. Rather, Ryk signaling may act as a significant modifier of FOXO3 activity.

### 2.3 FOXO3 binding sites are enriched for co‐regulator motifs

FOXO gene regulation involves other transcription factors that synergize with or antagonize FOXO proteins (Webb et al., 2013). The *in silico* motif analysis of FOXO3 binding sites in HD or C116 NSCs with or without Ryk silencing detected commonly enriched motifs such as Forkhead, as expected, Homeobox, Sp/KLF, ETS, E2F, Pou domain, PAS domain, JUN, zinc finger, TCF, C/EBP, and MEF (Figure S3). Regardless of Ryk silencing, Forkhead motifs showed a stronger enrichment and higher frequency in HD compared with C116 NSCs (Figure S3), consistent with higher FOXO3 occupancy (Figure 2c, middle panel), which also applied to other shared motifs (Homeobox, Sp/KLF, E2F, Pou domain). Some motifs (ASCL, bHLH, p53, SRF) appeared to be specifically enriched in HD NSCs (Figure S3), which could be due to better sampling of DNA fragments near FOXO3 binding sites owing to chromatin modifications in HD cells (Achour et al., 2015). Silencing Ryk did not alter co‐regulator motif profiles in either HD or C116 cells, except for Tbrain factor motifs as detected in HD cells with Ryk silencing (Figure S3), supporting a model in which Ryk signaling modulates FOXO3 gene regulation by altering the stoichiometry of the FOXO3/ß‐catenin/Ryk‐ICD complex (Figures 1 and 2).

### 2.4 FOXO3 binding sites overlap between human C116 and mouse NSCs

Some FOXO3 target families, *for example*, those responding to stress and proteotoxicity, may be conserved across species and cell types (Webb, Kundaje, & Brunet, 2016) while others are not conserved (Webb et al., 2016). We compared FOXO3 binding sites in human C116 NSCs with those previously reported in mouse NSCs (Webb et al., 2013) as both studies similarly analyzed F3Ts. To this end, we considered the best human orthologs of mouse genes bearing FOXO3 binding sites. A significant overlap was detected (20.25%, 446 genes, *p* = 3.15 × 10^−8^), suggesting that FOXO3 gene regulation and functions have common features in human and mouse NSCs (Table S3, Figure S4).

### 2.5 F3T reprogramming in human HD NSCs implicates regulators of cell senescence

We prioritized F3Ts based on the strength and convergence of the reprogramming effects across F3T‐INs categories, including targets that are (a) conserved between HD and C116 NSCs, (b) reprogrammed in a Ryk‐independent manner (no change upon Ryk silencing in HD cells, regardless of status upon Ryk silencing in C116 cells) and (c) dependent on Ryk in HD cells (target status corrected to normal upon Ryk silencing, regardless of status upon Ryk silencing in C116 cells).

First, we performed Enrichr analyses, retaining the top 1‐3 annotations for pathways and ontologies. Conserved F3T‐INs (Table S2/Sheet 3: 82 genes) are enriched for TNF signaling (KEGG pathway: *p* = 8.94 10^−05^) and Positive regulation of oxidative stress‐induced neuron death (Gene Ontology Biological Process (GOBP): *p* = 1.84 10^−05^). F3T‐INs lost or gained in HD NSCs with no effect of Ryk silencing (Table S4: 214 genes) are enriched for PI3‐AKT signaling (KEGG Pathway 2016: *p* = 3.23 10^−04^) and Positive regulation of protein autoubiquitination (GOBP: *p *= 1.88 10^−05^). Finally, Ryk‐dependent F3T‐INs in HD NSCs (Table S5: 111 genes) showed no enrichment for pathways, but displayed low‐significance enrichment for the GOBPs Positive regulation of JNK cascade (*p* = 1.88 10^−04^) and Regulation of cell cycle (*p* = .0054).

To enhance the precision of F3T prioritization, we performed network analysis using F3T‐INs as seeds for extracting high‐confidence networks from the STRING database (Szklarczyk et al., 2015). This analysis highlighted interconnected F3T‐INs that implicate core FOXO3 functions such as for example transcription, translation, and protein quality control (Webb et al., 2016). These F3Ts included those in the conserved (Figure S5A) or reprogrammed (Figure S5B,C) group(s) that belong to Wnt, Hippo/TGF‐ß (e.g., LATS2), Toll‐like receptor and mTOR signaling. This analysis also highlighted Ryk‐independent and Ryk‐dependent F3Ts that in HD NSCs are relevant to neuron differentiation, synaptic function, and cell cycle (Figure S5B,C). In the Ryk‐dependent group, network analysis (here, F3T‐INs showing the strongest regulation by FOXO3 and at least 3 out of 4 classes of F3T‐INs connected to the same node) predicted that, in HD NSCs, FOXO3 (a) no longer activates *CDKN2AIP* (also known as CARF), a co‐activator of p14^ARF^ (b) activates *SERTAD1* (also known as p34(SEL1)), an inducer of neuronal apoptosis when in excess that renders CDK4 resistant to inhibition by p16^INK4a^ (Li et al., 2005) and (c) represses *ETS2*, a transcription factor that positively regulates p16^INK4a^ expression (Ohtani et al., 2001) and a F3T‐IN‐KD gene. Together, these FT3 changes suggest suppression of the *CDKN2A* locus, particularly the p16^INK4a^ segment, in HD NSCs.

### 2.6 FOXO3 represses ETS2 expression in human HD NSCs

**FIGURE 3:**
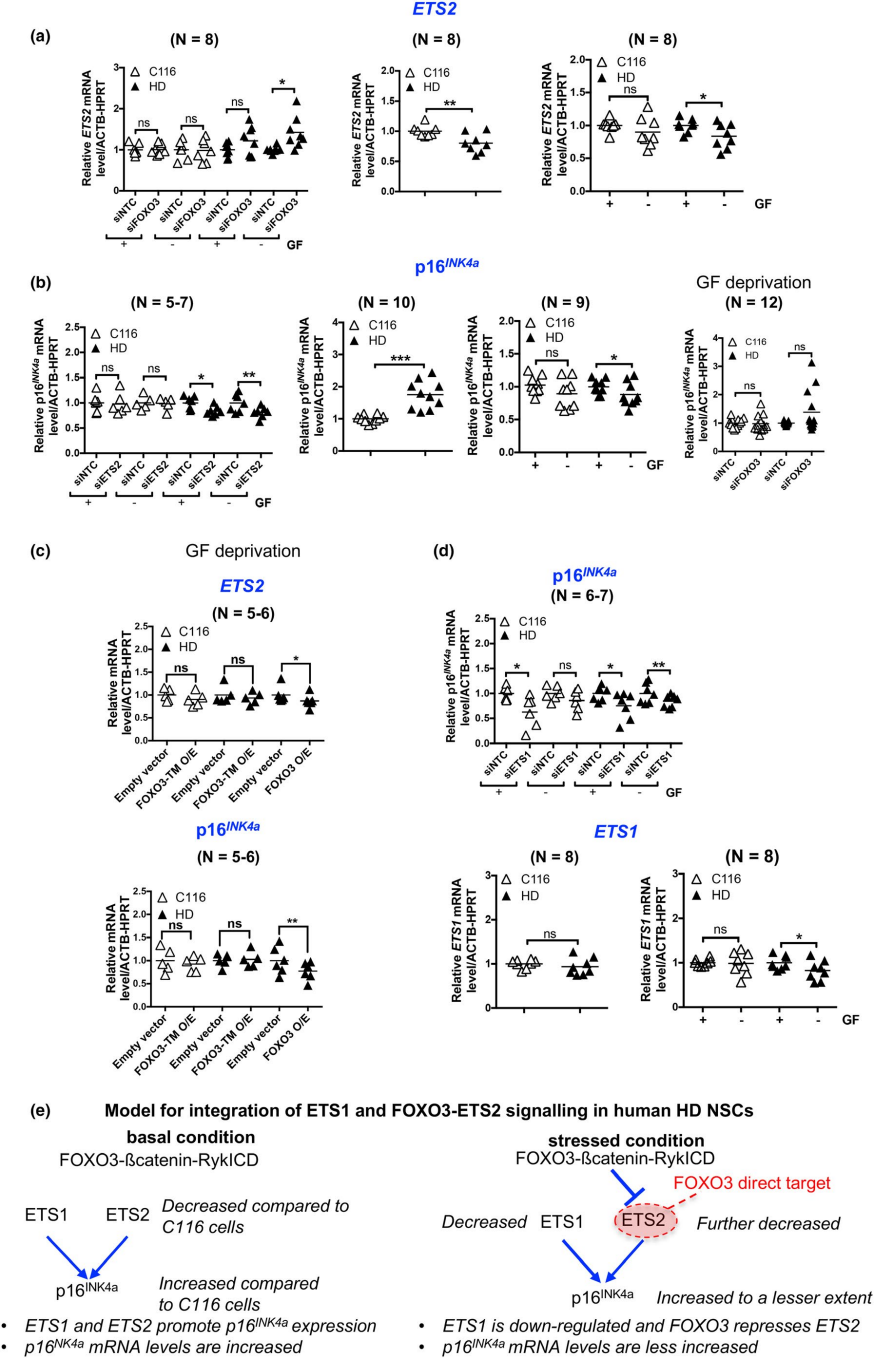
Gene expression analyses in human NSCs. The mRNA levels are normalized to cells treated with nontargeting control (NTC) siRNAs (siRNA tests) or to C116 cells or cells without growth factor (GF) deprivation (other experiments). ns, not significant. (a) *ETS2* mRNA levels are increased by *FOXO3* reduction in HD NSCs subjected to GF deprivation with no effect detected in basal conditions nor in normal *HTT* cells (left panel: *p < .05). *ETS2* mRNA levels are decreased in HD NSCs (middle panel: **p < .01). GF deprivation does not change *ETS2* mRNA levels in C116 and decreases *ETS2* mRNA levels in HD NSCs (right panel: *p < .05). (b) *p16^INK4a^* mRNA levels are decreased by *ETS2* reduction in HD NSCs in basal conditions and in cells subjected to stress with no effect detected in normal *HTT* cells (left panel: *p < .05, **p < .01). *p16^INK4a^* mRNA levels are increased in HD NSCs (middle left panel: ***p < .001). GF deprivation does not change p16^INK4a^ mRNA levels in C116 NSCs and decrease *p16^INK4a^* mRNA levels in HD NSCs (middle right panel: *p < .05). *p16^INK4a^* mRNA levels tend to be increased by FOXO3 knockdown in HD NSCs subjected to GF deprivation (right panel: not significant with *p* = .0736). (c) ETS2 and *p16^INK4a^* mRNA levels are decreased by overexpression of FOXO3, but not that of FOXO3‐TM, in human HD NSCs subjected to GF deprivation. The mRNA levels are normalized to cells treated with empty vector. *p < .05 and **p < .01. (d) *p16^INK4a^* mRNA levels are decreased by *ETS1* reduction in C116 NSCs in basal conditions and in HD NSCs in both basal and stress conditions (upper panel: *p < .05, **p < .01). *ETS1* mRNA levels are unchanged in HD compared with C116 NSCs (lower left panel). GF deprivation does not change *ETS1* mRNA levels in C116 NSCs and decreases *ETS1* mRNA levels in HD NSCs (lower right panel: *p < .05). (d) Working model for effect of FOXO3 target reprogramming on the ETS2‐p16^INK4a^ pathway

We performed validation studies of FOXO3 regulation of *SERTAD1*,*ETS2*, and *CDKN2AIP* in human NSCs subjected to stress. *FOXO3* silencing (Figure S6A) increased F3T‐IN‐KD *ETS2* mRNA levels in stressed HD NSCs, an effect not detected in unstressed HD and C116 NSCs (Figure 3a). Thus, *ETS2* is negatively regulated by FOXO3 in HD NSCs in response to stress, which could partially explain the down‐regulation of *ETS2* in these cells (Figure 3a, middle and right panels). In addition, *FOXO3* silencing decreased F3T‐IN *CDKN2AIP* mRNA levels in stressed C116 NSCs (Figure S7A). Thus, *CDKN2AIP* is a positively regulated F3T which is lost in HD cells and could decrease p14^ARF^ activity in these cells, potentially promoting cellular vulnerability (Wadhwa, Kalra, & Kaul, 2017). However, *CDKN2AIP* is upregulated in HD NSCs (Figure S6A, middle panel), which could increase the activity of p14^ARF^, a gene also upregulated in HD NSCs (see below), and promotes cellular resistance in a FOXO3‐independent manner. In contrast, the positive regulation of *CDKN2AIP* by FOXO3 in C116 NSCs could partially explain the increase of *CDKN2AIP* expression upon cell stress (Figure S7A, right panel). Finally, *FOXO3* silencing did not change F3T‐IN *SERTAD1* mRNA levels in HD NSCs (Figure S6B, left panel). Additionally, *SERTAD1* mRNA levels are slightly decreased in HD NSCs under basal conditions (Figure S7B, middle panel), with no change observed in stressed cells (Figure S7B, right panel). Thus, SERTAD1 does not appear to regulate stress response in HD NSCs.

### 2.7 *ETS2* positively regulates *p16^INK4a^* expression in human HD NSCs

ETS2 positively regulates *p16^INK4a^* expression in human fibroblasts (Ohtani et al., 2001). We asked whether p16^INK4a^ is under ETS2 regulation in HD NSCs. Silencing *ETS2* (Figure S6B) decreased *p16^INK4a^* mRNA levels in human HD NSCs under basal and stressed conditions, an effect not detected in C116 NSCs (Figure 3b), suggesting that ETS2 positively regulates *p16^INK4a^* expression in HD NSCs, regardless of stress exposure. Furthermore, *p16^INK4a^* mRNA levels increased in HD compared with C116 NSCs under basal conditions (Figure 3b, middle left panel), which declined upon cell stress (Figure 3b, middle right panel), possibly due in part to decreased *ETS2* elicited by FOXO3. FOXO3 knockdown tended to increase *p16^INK4a^* mRNA levels (Figure 3b, right panel, *p* = .073) but was not statistically significant, due to the transitive nature of this regulation via *ETS2*. The *CDKN2A* locus encodes p16^INK4a^ as well as 14^ARF^. Silencing *ETS2* (see Figure S6B) did not alter *p14^ARF^* mRNA levels in human HD NSCs (Figure S7C, left panel), suggesting that *ETS2* does not regulate *p14^ARF^* expression. The *p14^ARF^* mRNA levels were higher in HD compared with C116 cells under basal (Figure S7C, middle panel) and stressed (Figure S7C, right panel) conditions, which could promote cell cycle arrest, however in a FOXO3‐independent manner. ETS1 can also regulate p16^INK4a^ expression (Ohtani et al., 2001). Overexpression of FOXO3, but not that of FOXO3‐TM (a nonphosphorylatable mutant), decreased *ETS2* and *p16^INK4a^* mRNA levels in human HD NSCs subjected to growth factor deprivation (Figure 3c), suggesting that nucleo‐cytoplasmic shuttling of FOXO3 is required for repressing *ETS2* and for ensuring homeostasis of interactions with potential co‐repressors (van der Vos & Coffer, 2008), also suggesting that increased FOXO3‐binding to the *ETS2* promoter is part of the general increase of FOXO3 binding in HD NSCs. Silencing *ETS1* (Figure S6E) decreased *p16^INK4a^* mRNA levels in HD and C116 NSCs under basal conditions, an effect that was lost upon growth factor deprivation in C116 NSCs, but not HD NSCs (Figure 3d), suggesting that *ETS1* remains able to promote *p16^INK4a^* expression in HD NSCs under stressed conditions. *ETS1* mRNA levels were unchanged in HD compared with C116 NSCs (Figure 3d, lower left panel) and *ETS1* mRNA levels slightly declined in HD NSCs subjected to growth factor deprivation (Figure 3d, lower right panel). Thus, the ability of ETS1 to promote *p16^INK4a^* expression in HD NSCs may be dependent upon exposure to external stressors. Collectively, these results (Figure 3e) suggest that both the decreased expression of *ETS1* and FOXO3‐repression of *ETS2* may antagonize *p16^INK4a^* increase in human HD NSCs.

### 2.8 Prepatterned HD NSCs show cellular senescence features in striatal neurons

**FIGURE 4:**
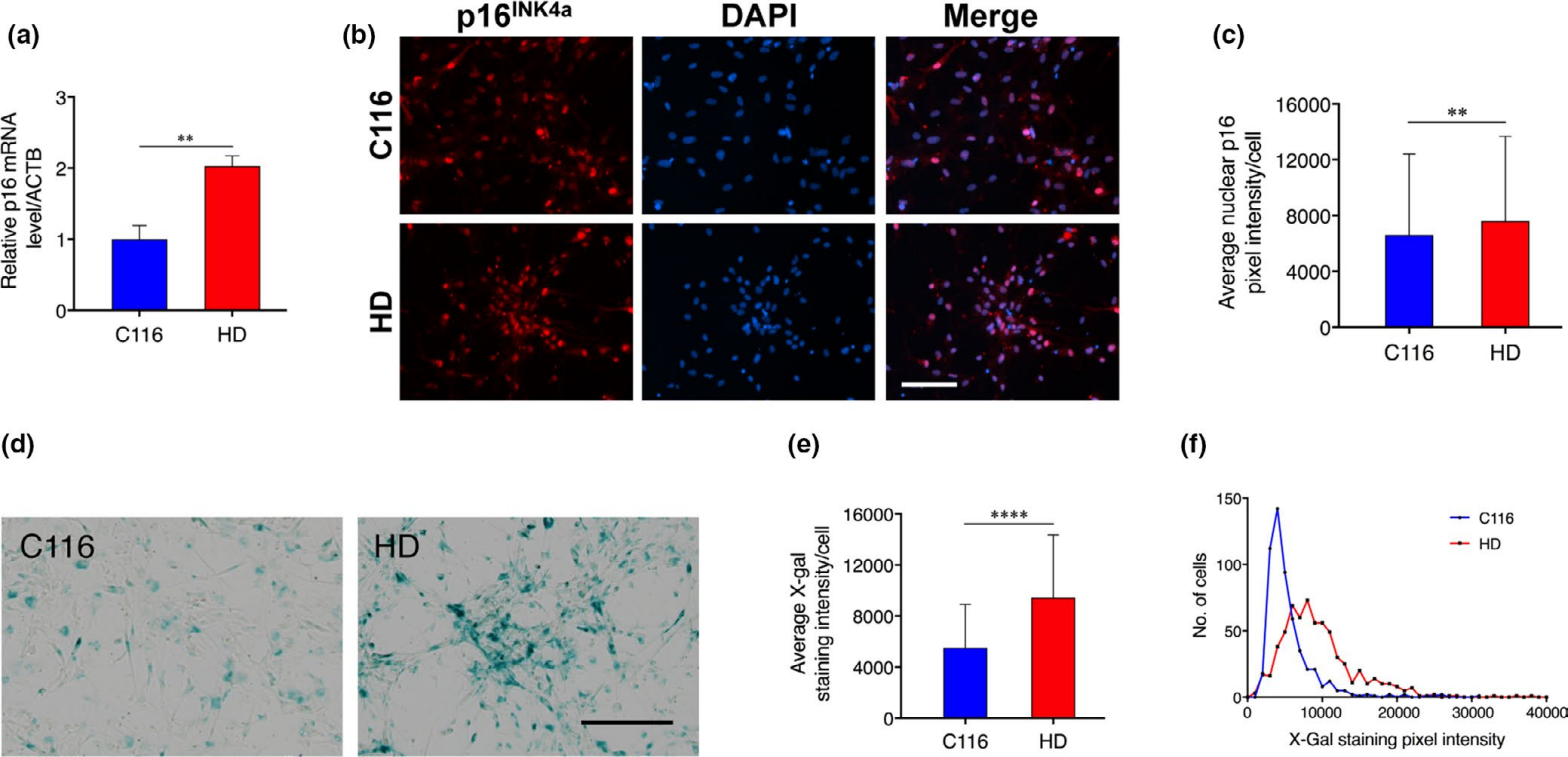
Human HD prepatterned NSCs show increase of p16^INK4a^ and of SA‐β‐gal activity. (a) p16^INK4a^ mRNA levels are increased in HD prepatterned NSCs. Data are mean ± *SD* (**p < .01), N = 3. (b) Representative images for modest p16^INK4a^ increase in HD NSCs. Scale bar in all panels: 100 µm. (c) Quantification of nuclear p16^INK4a^ pixel intensity for 532 C116‐NSCs and 1000 HD NSCs. Data are mean ± *SD* (**p < .01). (d) Representative images for increase of SA‐ß‐gal activity in HD NSCs. Scale bar in all panels: 200 µm. (e) Quantification of SA‐ß‐gal activity for 547 C116‐NSCs and 645 HD NSCs. Data are mean ± *SD* (****p < .0001). (f) Frequency distribution of SA‐ß‐gal signals for data shown in panel (e)

Given that p16^INK4a^ is a key effector of cellular senescence (Baker et al., 2016), we tested whether F3T reprogramming in HD NSCs might occur in the context of and respond to cellular senescence acquired in HD during neuronal differentiation. Using Activin A‐induced dorsoventral prepatterning, which efficiently directs striatal projection neuron differentiation of human iPSCs (Arber et al., 2015), we observed increase of *p16^INK4a^* mRNA and protein levels in HD compared with C116 prepatterned NSCs (Figure 4a,c). In addition, senescence‐associated ß‐galactosidase (SA‐ß‐gal) activity was more abundant in HD compared with C116 NSCs (Figure 4d,f). Importantly, in NSCs derived from additional nonisogenic HD (namely, ND41656 and ND42222) and control (namely, MIN08i‐33114.B and ND42241) iPSC lines, we observed robust increase in p16^INK4a^ expression (Figure S8A,B) and elevated SA‐ß‐galactosidase activity (Figure S8C), validating our results across multiple HD patients. We also tested for other markers of cellular senescence, including increased expression of *CDKN1A* encoding p21^CIP1^, *CDKN1B* encoding p27^KIP1^ and *MMP3* encoding a matrix metalloproteinase. Under basal conditions, *p21^CIP1^* mRNA levels were decreased (Figure S9A), *p27^KIP1^* mRNA levels were unchanged (Figure S9B) and *MMP*‐*3* mRNA levels were increased (Figure S9C) in HD compared with C116 NSCs. Thus, HD NSCs show increased levels of several markers of cellular senescence (p16^INK4a^, MMP‐3, SA‐ß‐gal).

**FIGURE 5:**
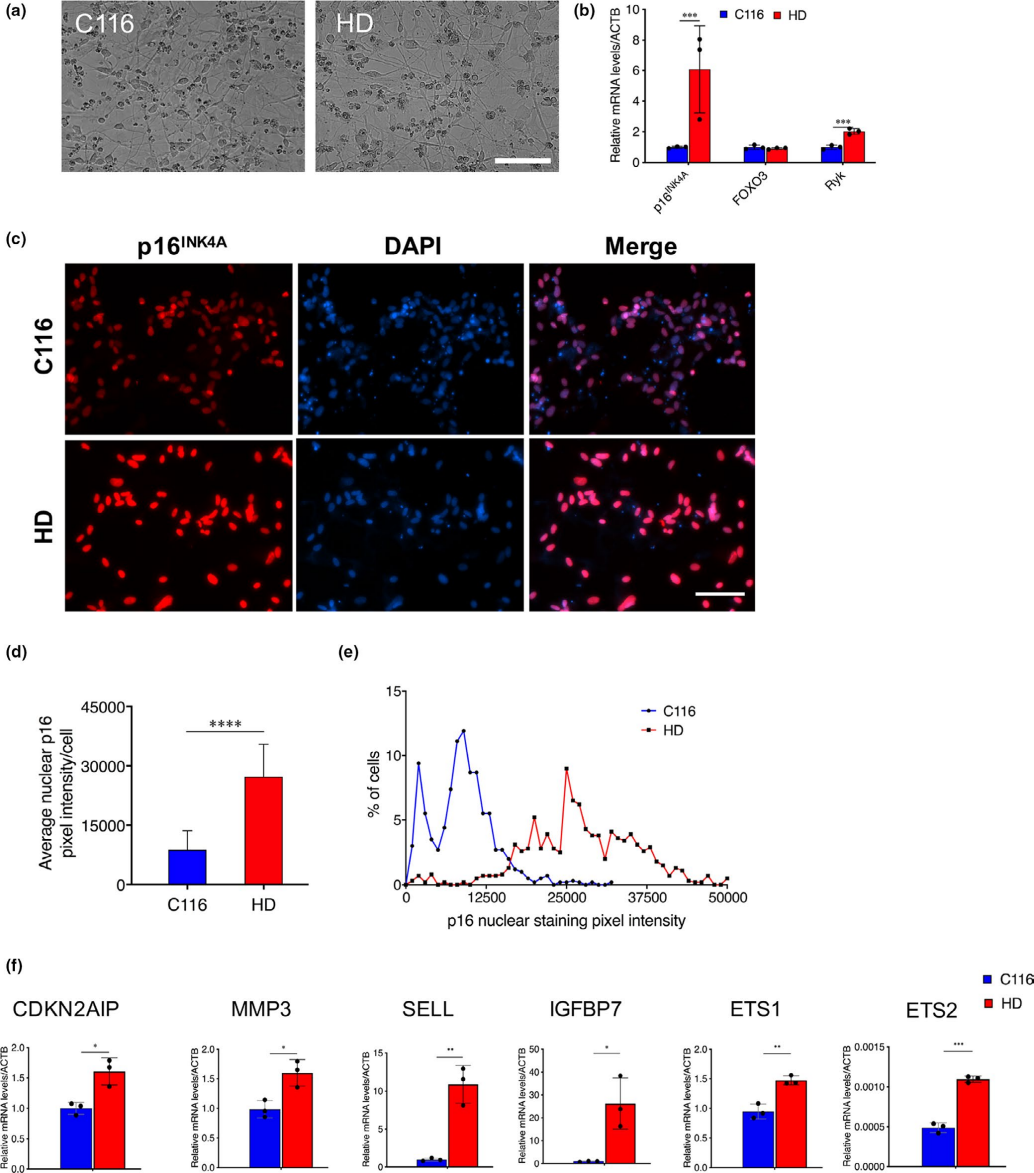
p16^INK4a^ expression is elevated in human HD MSNs. (a) Representative images of human NSC‐derived MSNs using defined enhanced media (Synaptojuice medium). (b) RT‐PCR analysis of p16^INK4a^, FOXO3, and Ryk in C116 and HD MSNs reveals modest increase of FOXO3 mRNA levels and robust increase of p16^INK4a^ and Ryk mRNA levels in HD MSNs. Data are mean ± *SD* (*p < .05, ***p < .001). *N* = 3. (c) Immunofluorescence analysis reveals dramatic increase of p16^INK4a^ in HD MSNs. Scale bar in all panels: 100 µm. (d) Quantification of nuclear p16^INK4a^ pixel intensity for *N* = 596 C116 NSCs and *N* = 609 HD NSCs. Data are mean ± *SD* (****p < .0001). (e) Frequency distribution of nuclear p16^INK4a^ signals for data shown in Panel (d). (e) RT‐PCR analysis of CDKN2AIP, MMP3, SELL, IGFBP7, EST1, and EST2 show increased mRNA levels in HD MSNs compared with C116 MSNs. Data are mean ± *SD* (*p < .05, **p < .01, ***p < .001). *N* = 3

Moreover, in HD differentiated MSNs derived from HD NSCs (Figure 5a), *p16^INK4a^* mRNA levels were strongly elevated, which was also true for Ryk mRNA levels (Figure 5b). This increase (~5‐fold) was greater in magnitude than that of *p16^INK4a^* mRNA levels in HD NSCs (~1.7‐fold; Figure 4a) and accompanied by increased p16^INK4a^ immunostaining (Figure 5c–e). We also found that other cellular senescence markers including CDKN2AIP, MMP3, SELL, and IGFBP7 were all upregulated in HD MSNs when compared with isogenic control C116 MSNs (Figure 5f). Further, ETS1 and EST2 are upregulated in HD MSNs compared with isogenic control (Figure 5f) further confirming the known role of ETS1 and EST2 in transcriptionally increasing *p16^INK4a^* (Ohtani et al., 2001). Human HD MSNs also showed decreased levels of nuclear HMGB1 (Figure S9D), which relocalizes to the extracellular space in senescent cells (Davalos et al., 2013), an effect not observed in HD NSCs. The size of a senescent cell increases when compared to nonsenescent cells. We found that human HD MSNs have an increase nuclear area when compared to C116 MSNs (Figure S9E). Together, these data suggest the differentiation of NSCs into striatal like neurons is accompanied by increasingly pronounced features of cellular senescence in HD.

### 2.9 *FOXO3* and *p16^INK4a^* oppositely modulate the vulnerability of human HD NSCs

**FIGURE 6:**
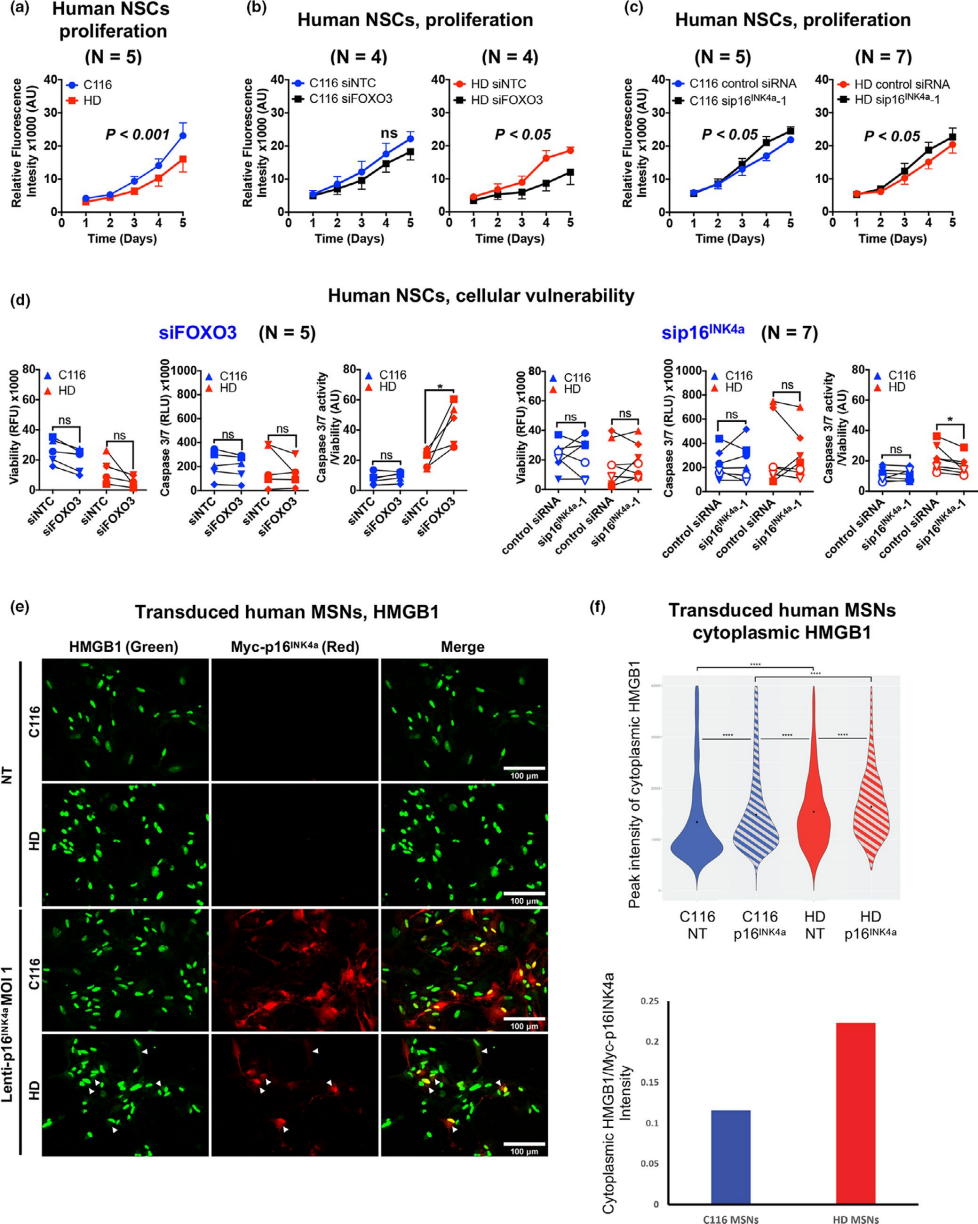
FOXO3 and p16^INK4a^ oppositely modulate the vulnerability of human HD NSCs. Significance was tested using two‐way ANOVA (panels a–c), paired *t* test (panels d) and Mann‐Whitney test (panel g). ns: not significant. (A) Human HD NSCs show reduced rates of cell growth. Data are mean ± *SEM*. (b) Reducing *FOXO3* does not alter the growth of C116 NSCs (left panel) and strongly reduces the growth of HD NSCs (right panel), with no change detected in *HTT* mRNA levels (see Figure S6F, left panel). Data are mean ± *SEM*. (c) Reducing *p16^INK4a^* slightly increases the growth of C116 (left panel) and HD (right panel) NSCs. Reducing *p16^INK4a^* does not alter *HTT* mRNA levels in HD NSCs (see Figure S5F, right panel). Data are mean ± *SEM*. (d) Reducing *FOXO3* increases the mortality of HD NSCs with no effect detected in C116 NSCs (left: *p < .05). Reducing *p16^INK4a^* decreases the mortality of HD NSCs with no effect detected in C116 NSCs (right: *p < .05). (e) Lenti‐myc‐*p16^INK4a^* transduction promotes nuclear release of HMGB1 in cytoplasm of HD and corrected (C116) MSNs. HD and C116 MSNs transduced for 4 days with lenti‐myc‐*p16^INK4a^* (red) were immunostained with HMGB1 (green). NT: transduction without myc‐*p16^INK4a^*. HMGB1 co‐localizes with the nucleus (DAPI), with low level in cytoplasm. The transduction with lenti‐myc‐*p16^INK4a^* significantly relocates HMGB1 into cytoplasm of HD and C116 MSNs (arrowhead). Scale bars: 100 µm. (f) Upper panel: The quantification of cytoplasmic HMGB1 pixel intensity shows a significant increase of nuclear HMGB1 release in HD vs. C116 MSNs and in HD vs. C116 MSNs following p16^INK4a^ overexpression (Wilcoxon ranked‐sum test: C116‐ *p16^INK4a^* vs. HD‐ *p16^INK4a^*, *p* = 6.1e‐22; C116‐*p16^INK4a^* vs. HD‐NT, *p* = 3.4e‐06; C116‐*p16^INK4a^* vs. C116‐NT, *p* = 1.8e‐23; HD‐*p16^INK4a^* vs. HD‐NT, *p* = 2.3e‐7; HD‐NT vs. C116‐NT, *p* = 5.6e‐37). Lower panel: data normalized against Myc‐p16^INK4a^ levels using the ratio (sum of HMGB1 intensity in ‘cells’ MOI 1/number of cells detected in cells MOI 1)/(sum of myc‐tag intensity in ‘cells’ MOI 1/number of cells detected in ‘cells’ MOI 1). The ratios show that HMGB1 relocalization is CAG‐repeat‐length‐dependent. C116‐p16^INK4a^: 1604 cells; C116‐NT: 1403 cells; HD‐p16^INK4a^: 879 cells; HD‐NT: 1792 cells

Next, we investigated whether FOXO3 activity in human HD NSCs might oppose the effects of p16^INK4a^. In cell growth assays, HD NSCs divided more slowly compared with C116 NSCs (Figure 6a). Reducing *FOXO3* (Figure S6F) retarded the growth of HD NSCs (Figure 6b, right panel) with no change detected in *HTT* expression (Figure S6H, left panel) and a trend (not significant) toward reduced growth of C116 NSCs (Figure 6b, left panel), suggesting that FOXO3 promotes the growth of human HD NSCs. Reducing *p16^INK4a^* (Figure S6G) slightly increased the growth of both HD (Figure 6C, right panel) and C116 (Figure 6c, left panel) NSCs, without changing *HTT* expression (Figure S6H, right panel), suggesting that *p16^INK4a^* normally restrains the growth of human NSCs, regardless of the *HTT* genotype. Together, these results suggest p16^INK4a^ does not significantly impact the dynamics of the NSC pool in HD.

In cell vulnerability assays, reducing *FOXO3* expression (Figure S5F) strongly potentiates the mortality of HD NSCs with no effect in C116 cells (Figure 6d). In contrast, reducing *p16^INK4a^* expression (Figure S6G) decreased the mortality of HD NSCs, with no effect detected in C116 cells (Figure 6d), suggesting that increased *p16^INK4a^* in human HD NSCs may have deleterious effects. Thus, FOXO3 transcriptional activity may tip the balance away from the detrimental effects of cell senescence features such as p16^INK4a^ increase on the homeostasis of the NSC pool in HD.

To further understand the role of *p16^INK4a^* in differentiated HD MSNs and cellular senescent‐like features, we transduced these cells with lentivirus expressing p16^INK4a^. We tested for HMGB1, an early responder to cellular senescence. We quantified cytoplasmic HMGB1 levels as a more sensitive measure of a senescent‐like phenotype compared with nuclear HMGB1 (Figure 6e). We found increased cytoplasmic HMGB1 basal levels in HD compared with C116 MSNs (Figure 6f). We also found increased cytoplasmic HMGB1 levels upon p16^INK4a^ transduction in HD and C116 MSNs (Figure 6f). These data suggest that p16^INK4a^ increase in HD MSNs may promote senescent‐like features in these cells.

### 2.10 *p16^INK4a^* mRNA levels are increased in the striatum of HD knock‐in mice

Analysis of published transcriptomic data (Langfelder et al., 2016) of the *Cdkn2a* locus (products p16^INK4^ and p19^ARF^) in *Hdh* mice shows a CAG‐repeat‐ and age‐dependent increase of *Cdkn2a* (Figure S10A). To assess *in vivo* relevance of senescence with p16^INK4a^ increase as observed in human HD iPSC‐derived cells, we used *Hhd*‐Q175 knock‐in mice. Given the lack of antibodies for a reliable study of p16^INK4a^ protein expression in mice (see Section 3), we tested for gene expression and we found that *p16^INK4a^* mRNA levels are strongly increased in the striatum of *Hdh*‐Q175 mice at 15 months of age, with a lesser increase detected in the cortex and no change in the cerebellum (Figure S10B). In HD postmortem caudate, published data show increase of *CDKN2A* relative to control (Agus, Crespo, Myers, & Labadorf, 2019) (Figure S10C). Further studies in mice and human tissues will be needed to confirm cellular senescence.

## 3 DISCUSSION

FOXO factors have widespread anti‐aging effects *via* the transcriptional regulation of stress response in multiple cell contexts (Martins et al., 2016; Salih & Brunet, 2008). Several cell maintenance mechanisms under FOXO control are affected in several NDs (e.g., mitochondrial homeostasis, proteostasis, autophagy, immune system, DNA repair). Understanding how FOXO gene regulation modulates brain cell maintenance in NDs may thus have important therapeutic implications. Although FOXO gene regulation has been studied in several cellular contexts (Webb et al., 2016), including in NSCs (Webb et al., 2013) and neurons (McLaughlin & Broihier, 2017), human FOXO targets in ND conditions are unknown as well as the biology of these targets in patient‐derived cells. Our data identify FOXO3 targets in human NSCs, suggesting a model in which human NSCs reprogram F3Ts in response to HD. Remarkably, this response takes place in the context of senescence that develops in these cells, involving the repression of the ETS2‐p16^INK4a^ axis, a mechanism that is part of the Ryk‐dependent element of F3T reprogramming. Our data suggest that Ryk signaling is a primary factor that modifies the FOXO3 target space. However, Ryk may signal through multiple mechanisms, including the canonical Wnt, PCP, and Ryk‐ICD pathways (Andre et al., 2012; Lyu, Yamamoto, & Lu, 2008; Tourette et al., 2014), and the effects of silencing Ryk on the F3T repertoire might also result from changes in pathways other than the Ryk‐ICD pathway. Nonetheless, in HD cells, our data suggest that pathways that signal onto FOXO3 such as Ryk/Ryk‐ICD signaling play a primary role in modifying the F3T repertoire, rendering FOXO3 able to fine‐tune the expression of key inducers of cellular senescence such as p16^INK4a^, whereas the increase of FOXO3 occupancy may primarily reflect the wide‐spread effect of HD on chromatin remodeling (Achour et al., 2015).

Stress response involves p16^INK4a^ in several stem cell types, during development or aging (D'Arcangelo, Tinaburri, & Dellambra, 2017; Oh, Lee, & Wagers, 2014). Human HD NSCs show senescence features, for example, p16^INK4a^ increase, that are increasingly pronounced as they differentiate into DARPP‐32 positive MSNs. Additionally, *p16^INK4a^* promotes the relocalization of HMGB1 to the cytoplasm, a senescence marker increased in human HD MSNs, in a CAG‐repeat‐dependent manner. These results suggest the HD brain might face a continuous cellular senescence process that affects neurogenesis and adult neurons. Transcriptional reprogramming by FOXO3 and repression of the ETS2‐p16^INK4a^ axis may be noticeably important to promote the robustness of the NSC pool as siRNA‐mediated reduction of *p16^INK4a^* expression decreased the mortality of HD NSCs. Our data thus suggest a model in which FOXO3 signaling can tip the balance away from cellular senescence in HD. Although siRNA‐mediated reduction of *p16^INK4a^* expression (about 70%) does not accurately recapitulate the reduction of *p16^INK4a^* expression (about 20%) that is elicited by the EST2‐p16^INK4a^ axis, our data indicate that reinforcing the outcome of FOXO3 activity in response to HD, that is, by further inhibiting p16^INK4a^ levels, may have therapeutic potential to avoid the harmful effects (maladaptation) of a chronic cellular senescence response in human HD neurons. Such an approach might be of interest for promoting adult neurogenesis in HD as adult‐born neurons may be depleted in the striatum of human HD brains (Ernst et al., 2014) and for targeting the detrimental consequences of neuronal senescence in other ND contexts. The ability of FOXO3 to tip the balance away from cellular senescence in response to CAG expansion in *HTT* could persist in adult neurons as the deregulation of senescence markers may be conserved from developmental to adult stages. Consistent with this, our data show that *p16^INK4a^* mRNA levels are strongly increased in the striatum of *Hdh* mice. Additionally, p16^INK4a^ is increased in HD NSCs differentiated into MSNs.

Our data suggest that neural and neuronal senescence could be set early in HD, a ND associated with chromatin remodeling (Achour et al., 2015), and has potential to be prosecuted in view of early drug trials (e.g., during prodromal disease). However, additional studies in the brain of HD mice and in human HD postmortem brains are needed to test for the relevance of senescence to HD. We attempted to test for p16^INK4a^ levels in the striatum of *Hdh* mice using p16^INK4a^ antibodies (i.e., MAS‐17142). However, we observed that MAS‐17142 recognizes mouse p16INK4a in a nonreliable manner (Western blot detection of a band presumably corresponding to p16^INK4a^ in the 60–70 kDa range, suggesting p16^INK4a^ oxidation/aggregation; detection of a nucleolus signal that looks like an aggregated signal in immunochemistry experiments), a problem formerly pointed for several antibodies claimed to properly recognize mouse p16^INK4a^.

Given the tight links between chromatin remodeling, NDs and cellular senescence (Achour et al., 2015; Criscione, Teo, & Neretti, 2016; Jakovcevski & Akbarian, 2012), our data raise the possibility that neural/neuronal senescence could be set early in NDs such as Alzheimer's and Parkinson's. Although senolytics may positively impact on brain activity in mouse models of NDs via removing senescent glial cells (Bussian et al., 2018; Zhang et al., 2019), they might have negative effects by removing neurons and neuronal connections that bear senescence features but retain a proper activity. Based on our findings, we hypothesize cell‐type‐specific strategies that can oppose specific detrimental effects of cellular senescence while preserving cellular homeostasis may be safer, particularly in early drug trials.

In conclusion, our data show that cellular senescence features, including increase of p16^INK4a^, develop during differentiation of human HD iPSC‐derived cells to persist in human HD MSNs. Our data suggest that FOXO3 may antagonize the progression of cellular senescence in ND conditions, repressing ETS2 in human HD NSCs, which reduces the expression of *p16^INK4a^*, in turn fine‐tuning stress response. These findings provide a rationale and target, early senescence‐like responses, to develop pro‐resilience approaches that may be useful for early intervention in HD and other NDs.

## 4 EXPERIMENTAL PROCEDURES

### 4.1 Cell culture

Human Embryonic Kidney 293 cells (HEK293T) were cultured in DMEM medium (Gibco), 10% FBS (Gibco) and 100U/ml penicillin and 100 μg/ml streptomycin (Gibco) at 37°C, 5% CO_2_. Human iPSCs derived from an HD patient (female—20 years old: 72Q/19Q) and their CAG‐corrected counterpart (21Q/19Q: C116) (An et al., 2012) were used. iPSCs were differentiated into NSCs (Ring et al., 2015). The differentiation into NSCs was tested by immunofluorescence using antibodies against the NSC markers Nestin (Sigma‐Aldrich, 1:200) and SOX1 (Sigma‐Aldrich, 1:50) and iPSC marker OCT3/4 (Pierce antibodies, 1:500). Differentiation into NSCs across all experiments was at least 98%. The iPSC lines were verified for genome integrity prior to performing experiments using multi‐color FISH analysis carried out by Applied Stemcell Inc. To generate prepatterned Activin A NSCs, the NSCs generated using above protocol were consistently maintained in 25 ng/ml Activin A (Peprotech) after EB stage starting at day 10.

Nonisogenic HD and control iPSC lines ND41656 (CAG 57), ND42222 (CAG 109), ND42241 were obtained from Coriell Repository, and MN08i‐33114.B line from WiCell. NSC lines were generated using PSC neural induction medium (Life Technologies) as per instructions in the manual. Briefly, iPSCs cultured in mTeSR were harvested using 1 mg/ml collagenase. The colonies were transferred to a 60 mm dish coated with Matrigel (1:60 dilution, BD Biosciences) and cultured in PSC neural induction medium supplemented with 1 µM LDN‐193189 and 10 µM SB431542 for 7 days to induce neuroepithelial fate. These cells were then harvested and expanded in neural expansion medium (PSC neural induction medium and DMEM/F12 medium (1:1), 100 U/ml penicillin and 100 μg/ml streptomycin, and 2 mM l‐Glutamine) supplemented with 25 ng/ml bFGF.

### 4.2 Analysis of the FOXO3/ß‐catenin/Ryk‐ICD complex

The methods used for protein co‐immunoprecipitation and deletion mapping assays are described in the Appendix S1.

### 4.3 Analysis of FOXO3 gene regulation

The methods used for analyzing FOXO3 targets in human HD NSCs are described in the Appendix S1.

### 4.4 Analysis of cellular senescence

The methods used for testing cellular senescence are described in the Appendix S1.

### 4.5 Differentiation of human NSCs into MSNs

The 60 mm dishes or 6‐well plates were coated with 100 μg/ml poly‐d‐lysine (Sigma‐Aldrich, P6407) followed by Matrigel (1:60, Corning) coating. NSCs were plated and cultured in NPM. When confluent, NSCs were treated with Synaptojuice A medium for 1 week followed by Synaptojuice B medium for 10 d at 37°C (Kemp et al., 2016). 25 ng/ml Activin A was added to both Synaptojuice A and Synaptojuice B media. Half media change was performed every 2 days. The resulting MSNs were characterized by immunofluorescence using antibodies (1:50–1:100) against the following: ß‐III‐tubulin (SCBT, sc‐80005), DARPP‐32 (SCBT, sc‐11365), Calbindin D‐28K (Sigma‐Aldrich, C9848), GABA (Sigma‐Aldrich, A2052), MAP2 (EMD Millipore, AB5622), and c‐myc antibody (SCBT, sc‐40). MSNs labeled positively for these markers and transductions. DARPP‐32 expression was also determined by RT‐PCR. Lentivirus transduction was performed with myc‐p16^INK4a^ from Origene (RC220937L1V) using a multiplicity of infection (MOI) of 1 and transduction without myc‐p16^INK4a^ as a control. After 4 days of Synaptojuice B MSN differentiation, lentivirus was applied into Synaptojuice B without CHIR 99021 (Tocris 4423). MSNs were transduced with virus for 4 days.

### 4.6 Cell transfection and cellular assays

The methods used for transfection and for testing cellular proliferation and vulnerability are described in the Appendix S1.

### 4.7 Statistics

Statistics were performed using Student's *t* tests or two‐way ANOVA. All experiments were repeated at least three times. *p* < .05 was considered significant. Statistics used for genomic data analysis, overlap analysis, and biological content analysis are described in the Appendix S1.

## CONFLICT OF INTEREST

None declared.

## AUTHOR CONTRIBUTIONS

JV performed stem cell work, genomic experiments, analyzed the data, and wrote the manuscript. FF performed biochemical analysis, cell proliferation, and vulnerability assays; analyzed the data; and wrote the manuscript. SN and AG performed senescence experiments. JD performed mouse studies. MF performed gene expression and cellular studies and analyzed the data. SSN and F‐XL performed bioinformatic and statistical analysis. SN, K‐TT, CGA, AL‐R, KLM, and NZ performed cell differentiation and senescence experiments. MV analyzed the data and helped write the manuscript. JC provided essential reagents and advice for senescence tests and edited the manuscript. LME contributed human cell differentiation protocols, designed the research, analyzed the data, and wrote the manuscript. CN conceived and designed the research, analyzed the data, and wrote the manuscript.

## Data Availability

RNA‐seq and ChIP‐seq data are available at GSE109873, subseries GSE109871, GSE109872, and GSE109869.
