# Supplementary figures and images for: FOXO3 targets are reprogrammed as Huntington's disease neural cells and striatal neurons face senescence with p16INK4a increase
Source: Aging Cell. 2020 Nov 6;19(11):e13226. doi: 10.1111/acel.13226 (PMC7681055; doi:10.1111/acel.13226)

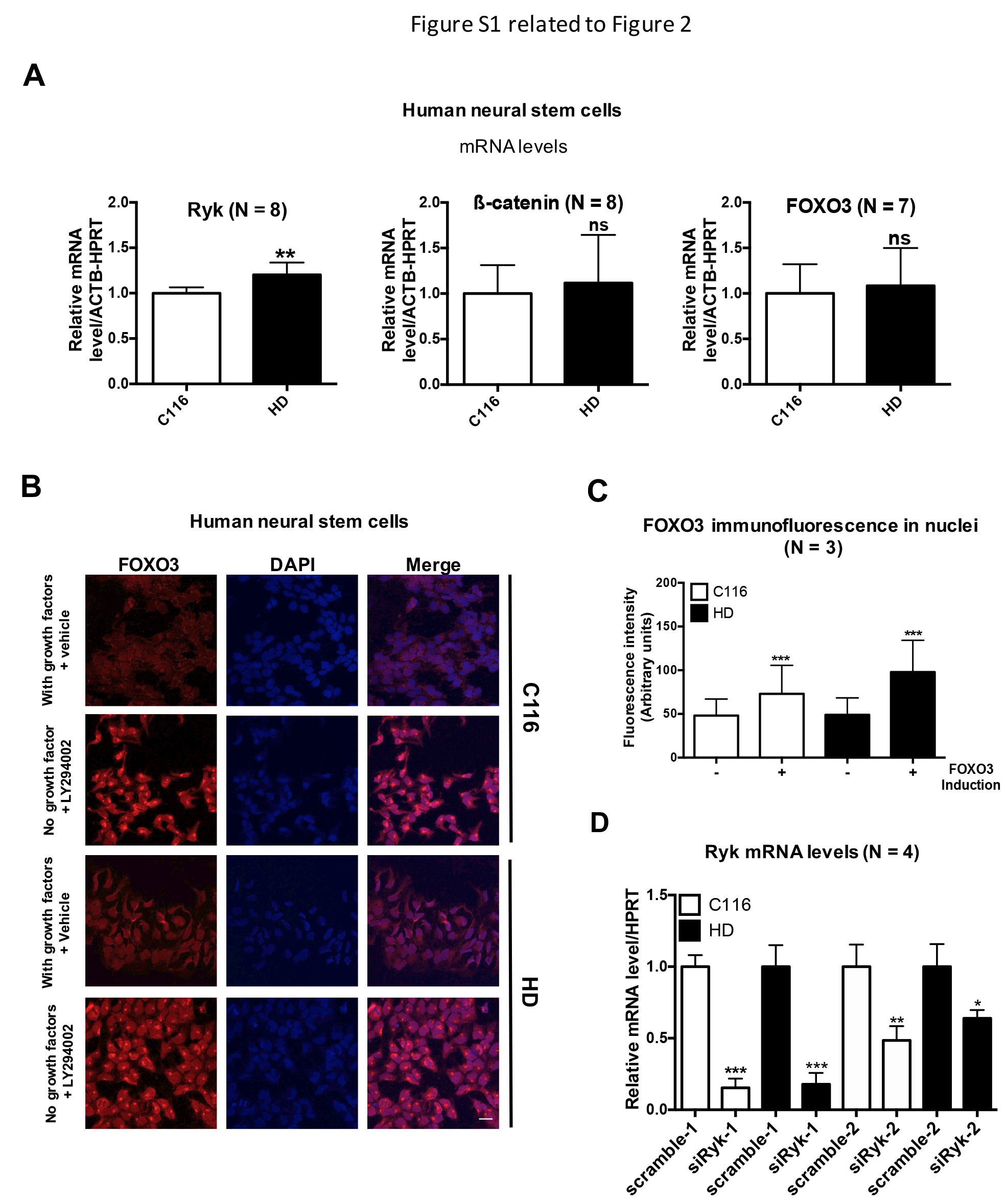

Supplement: Supplementary file 1 [file ACEL-19-e13226-s001.tif]

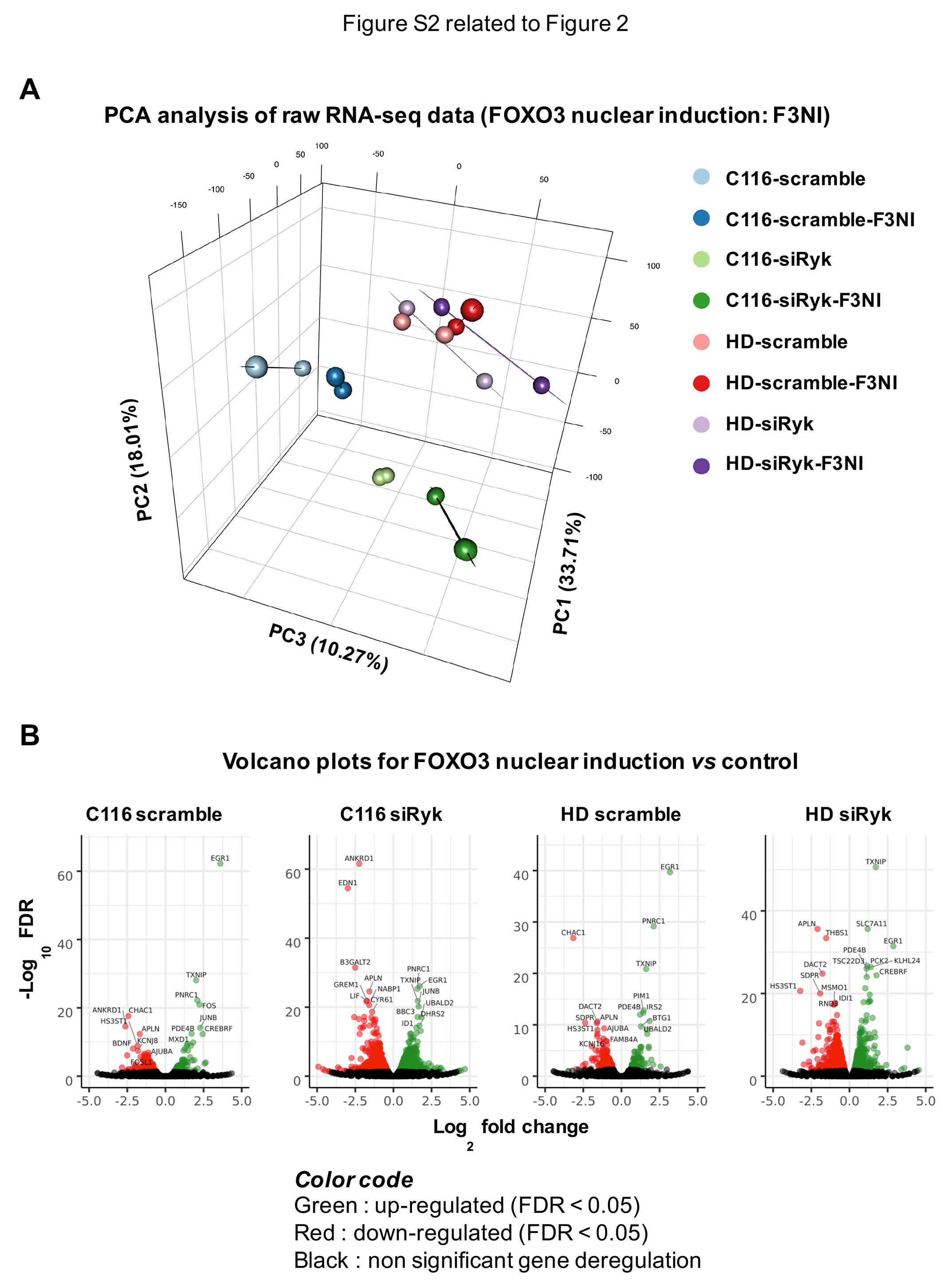

Supplement: Supplementary file 2 [file ACEL-19-e13226-s002.tif]

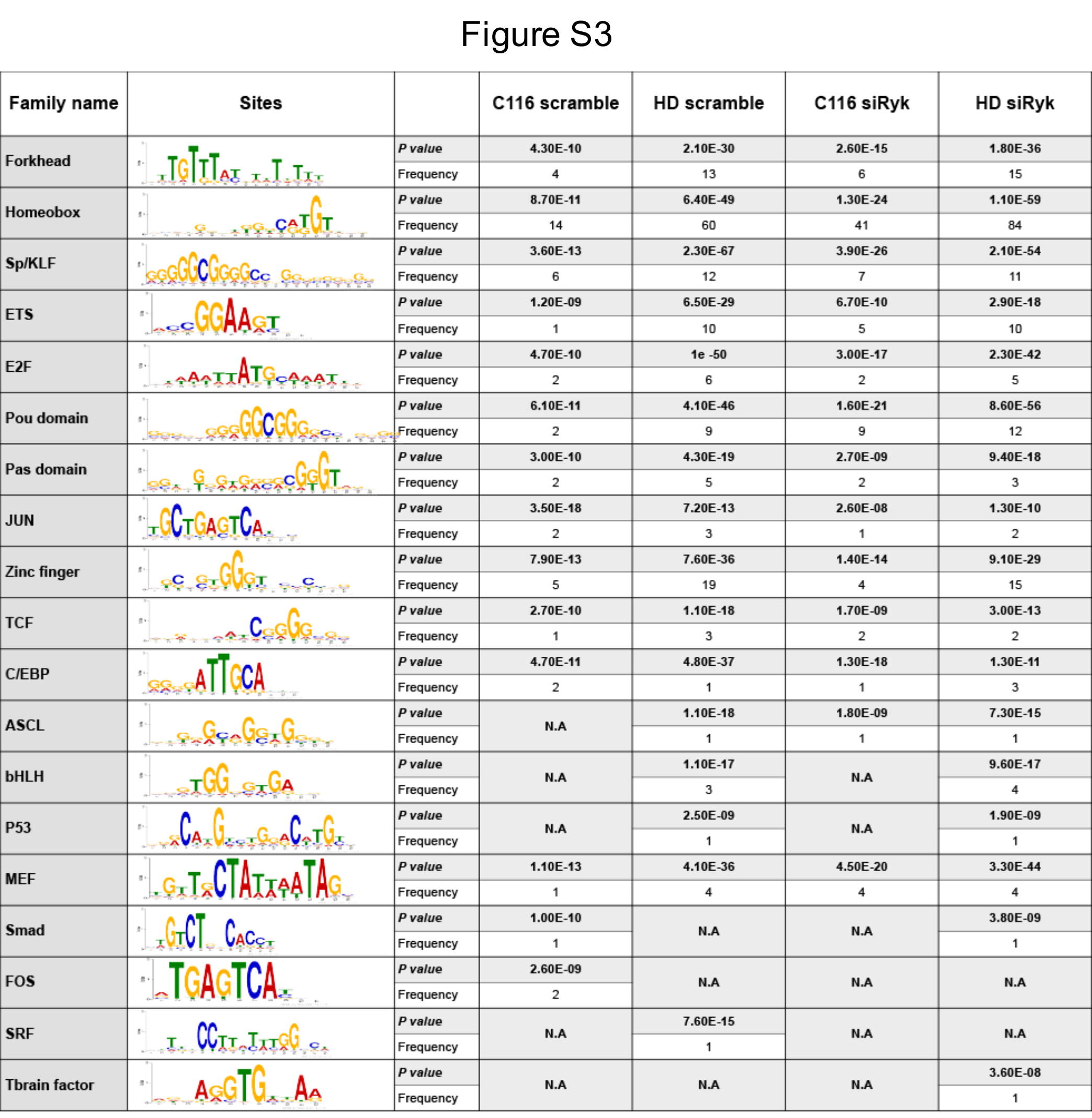

Supplement: Supplementary file 3 [file ACEL-19-e13226-s003.tif]

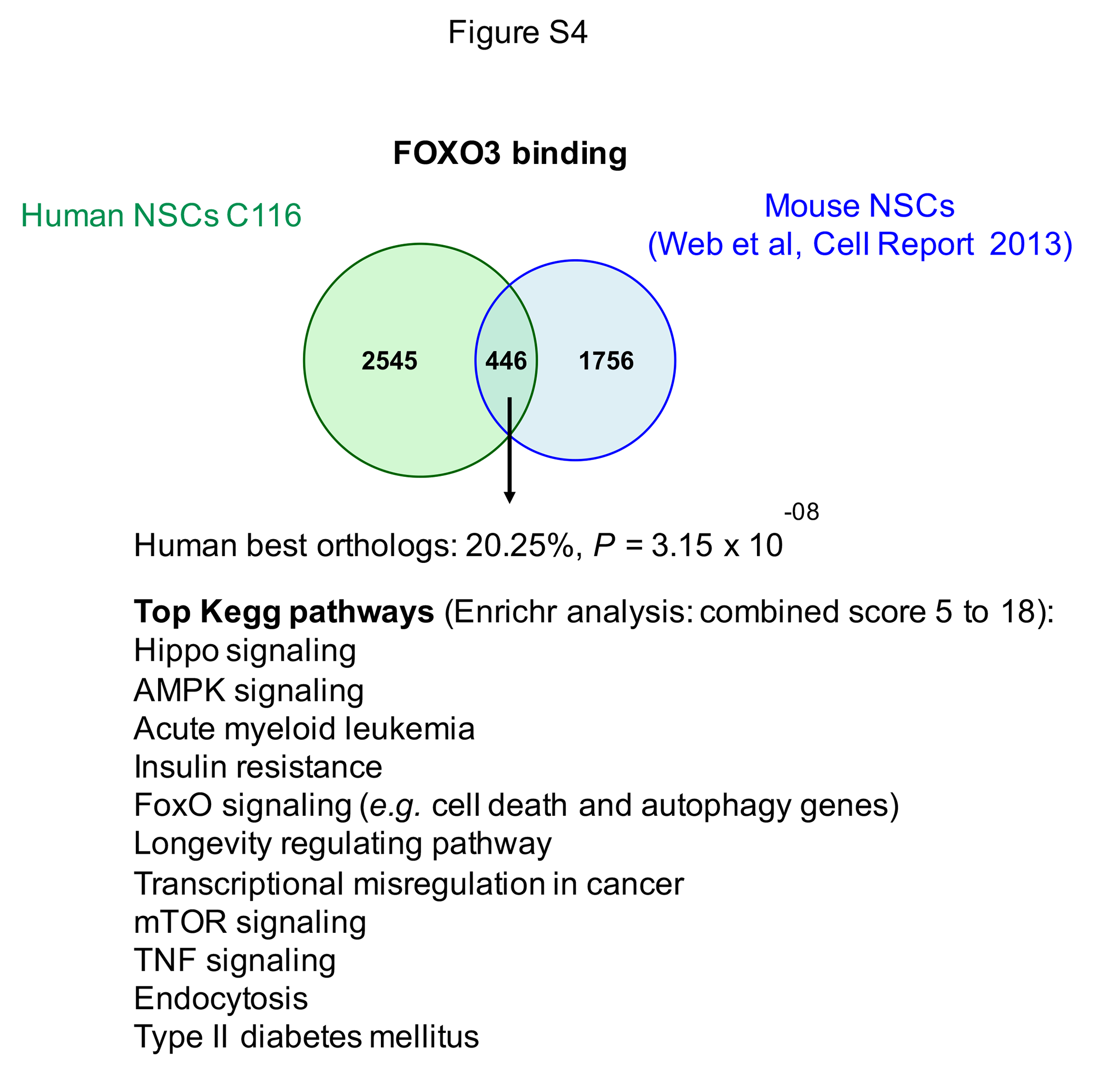

Supplement: Supplementary file 4 [file ACEL-19-e13226-s004.tif]

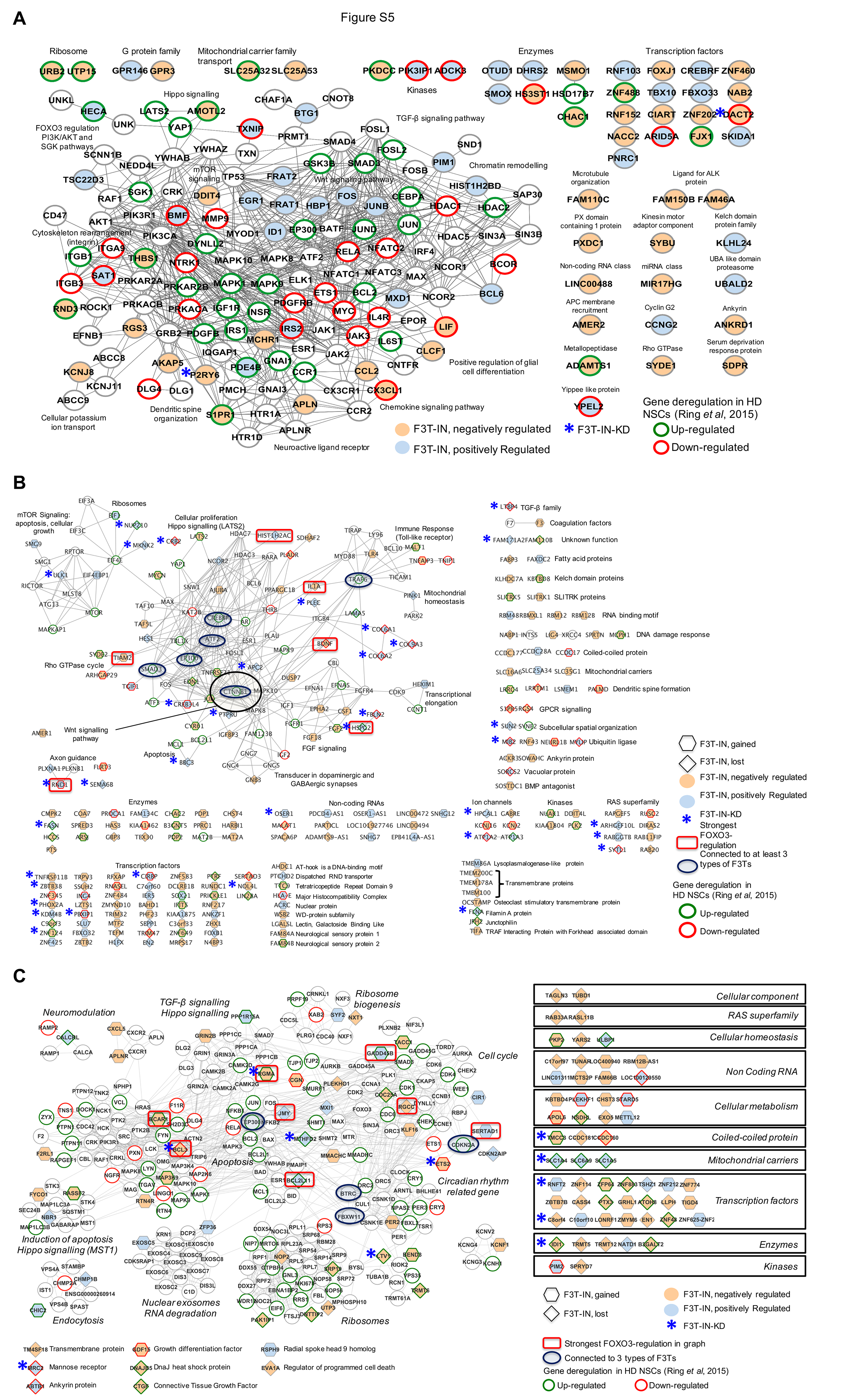

Supplement: Supplementary file 5 [file ACEL-19-e13226-s005.tif]

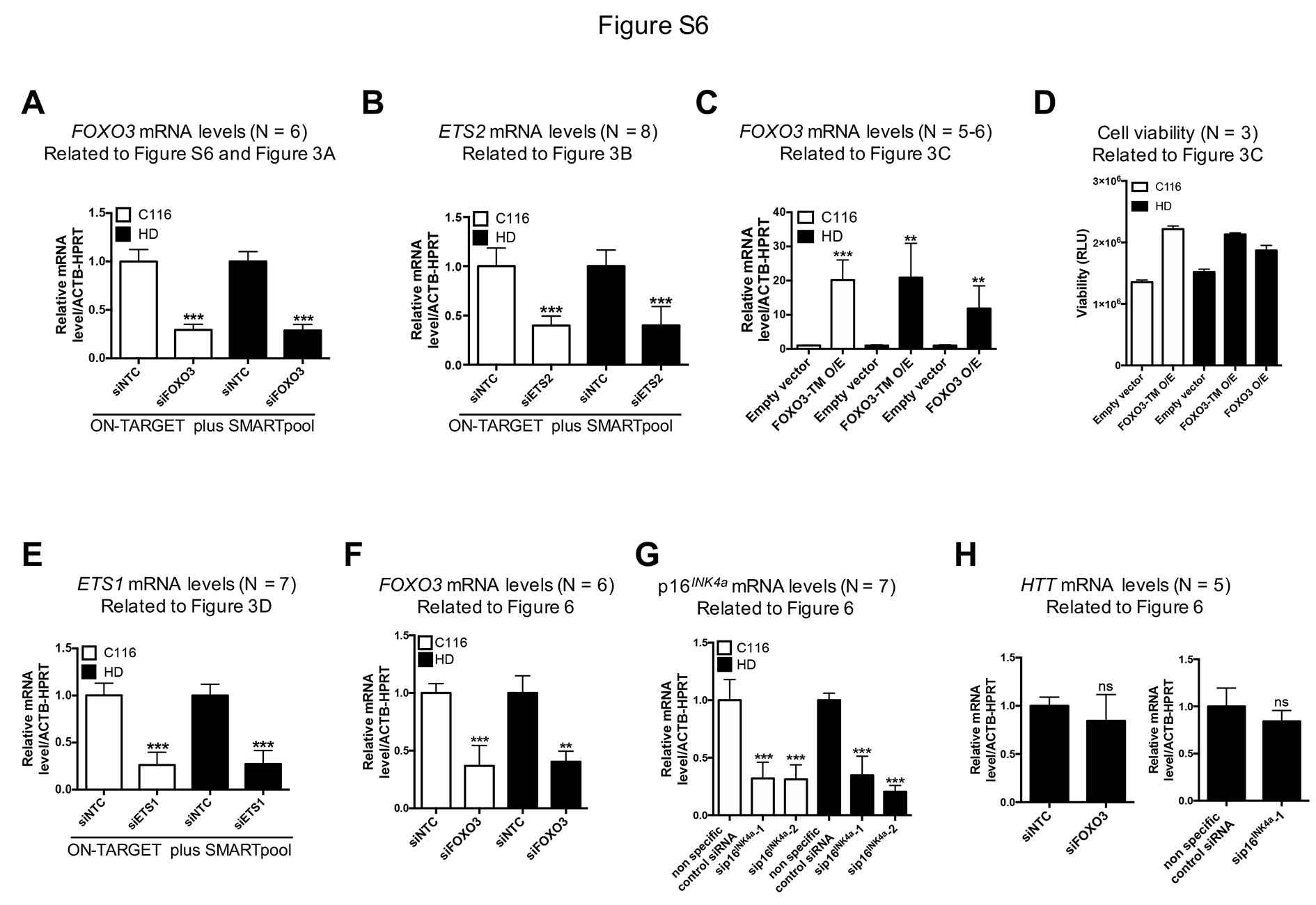

Supplement: Supplementary file 6 [file ACEL-19-e13226-s006.tif]

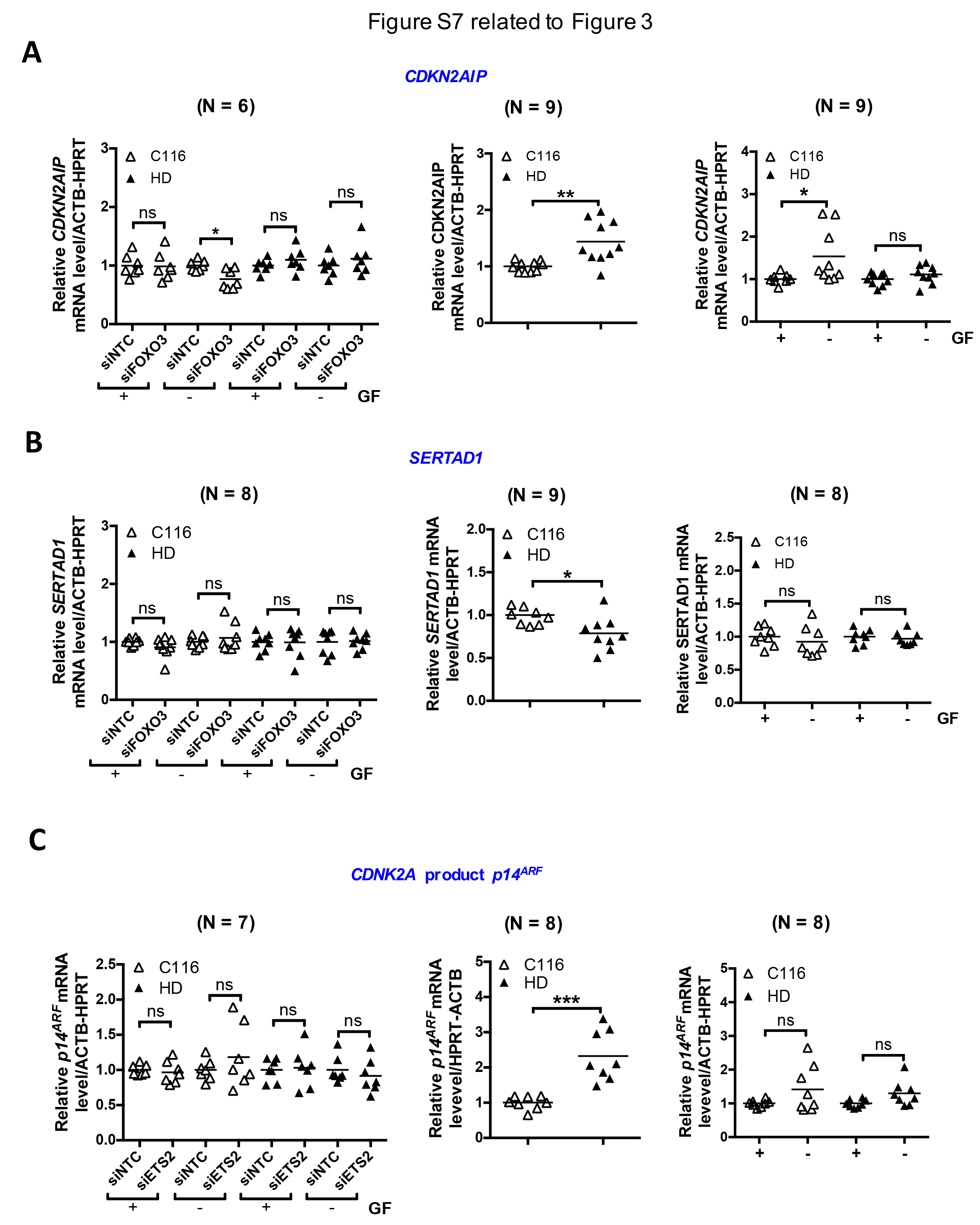

Supplement: Supplementary file 7 [file ACEL-19-e13226-s007.tif]

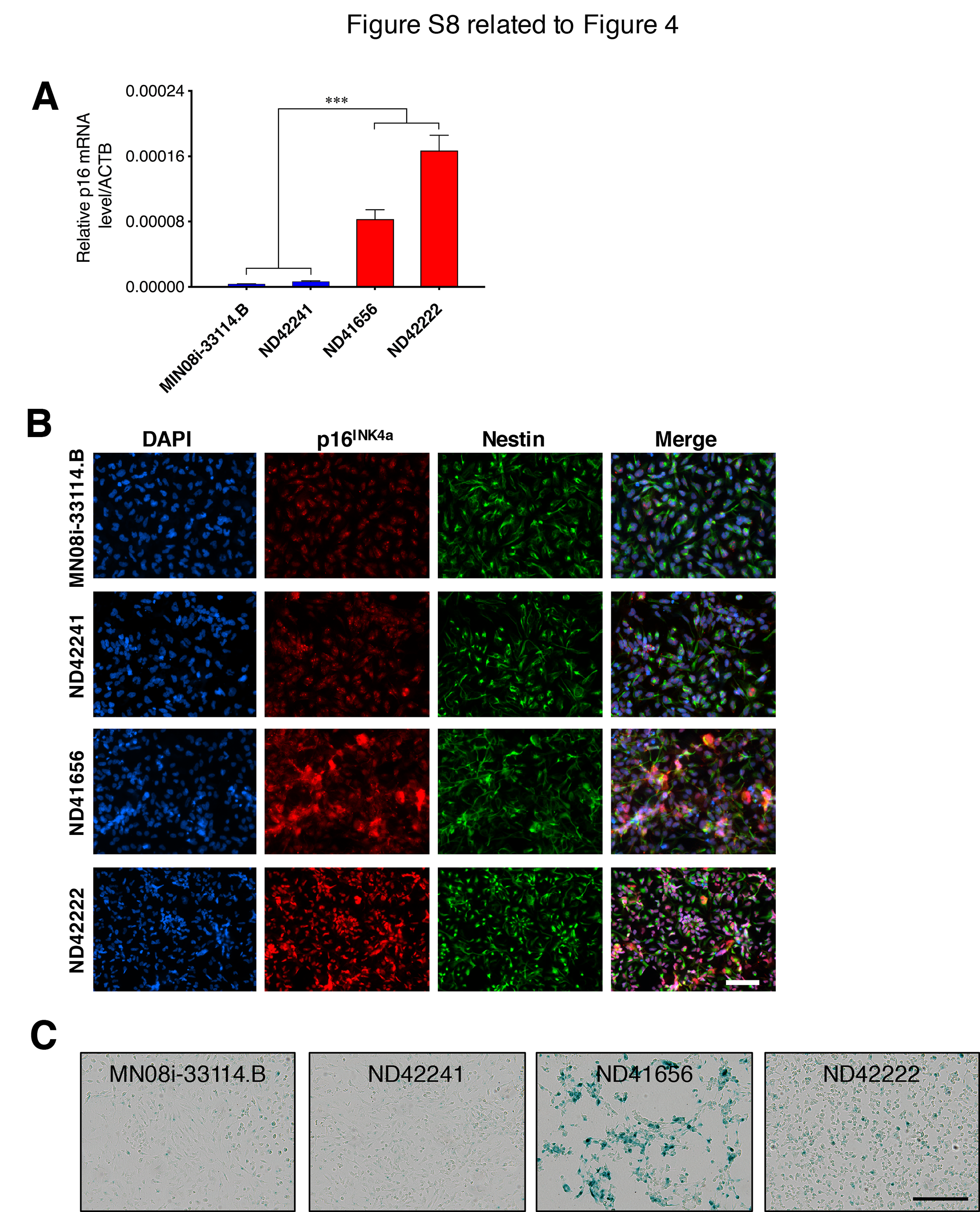

Supplement: Supplementary file 8 [file ACEL-19-e13226-s008.tif]

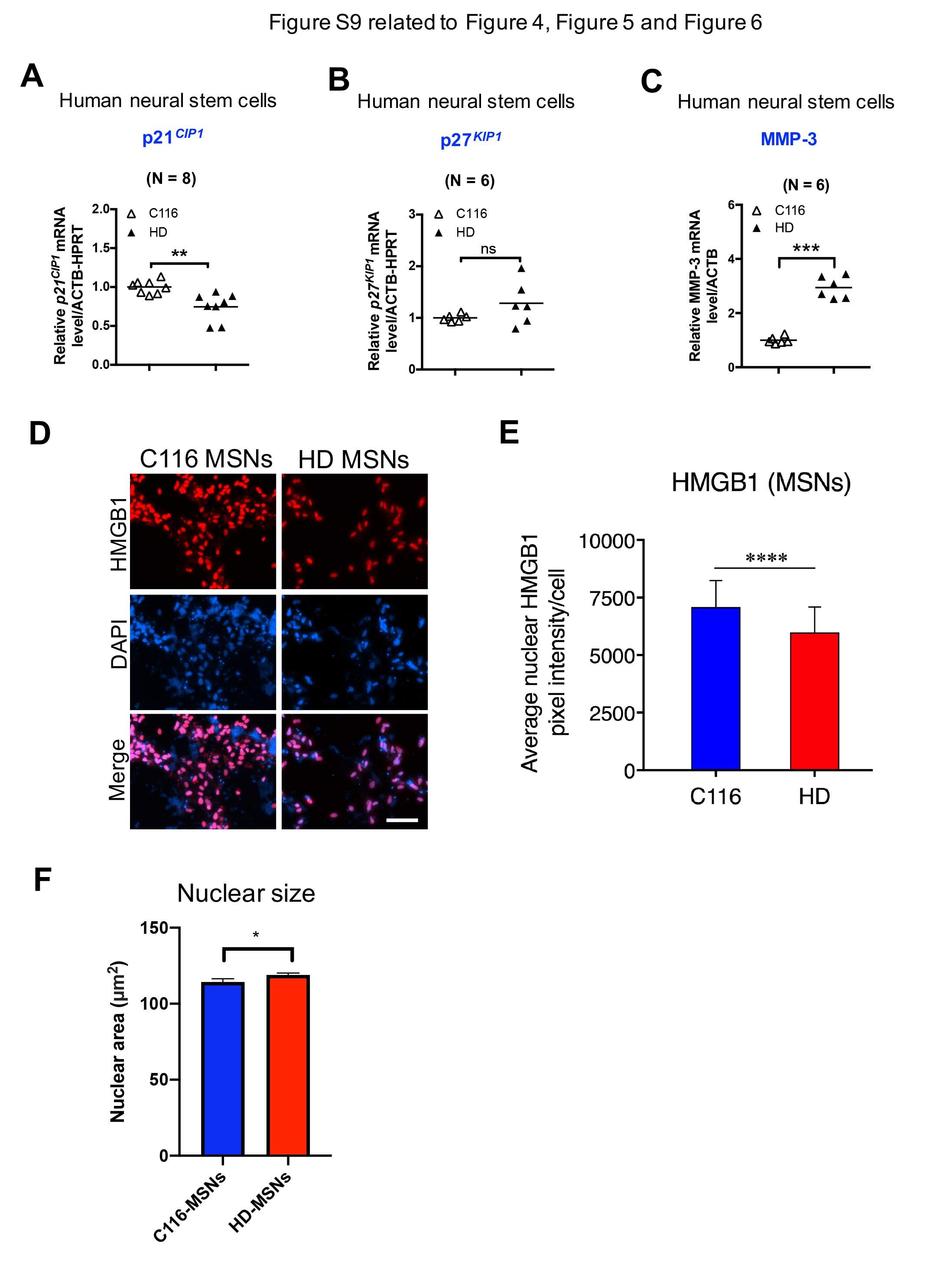

Supplement: Supplementary file 9 [file ACEL-19-e13226-s009.tif]

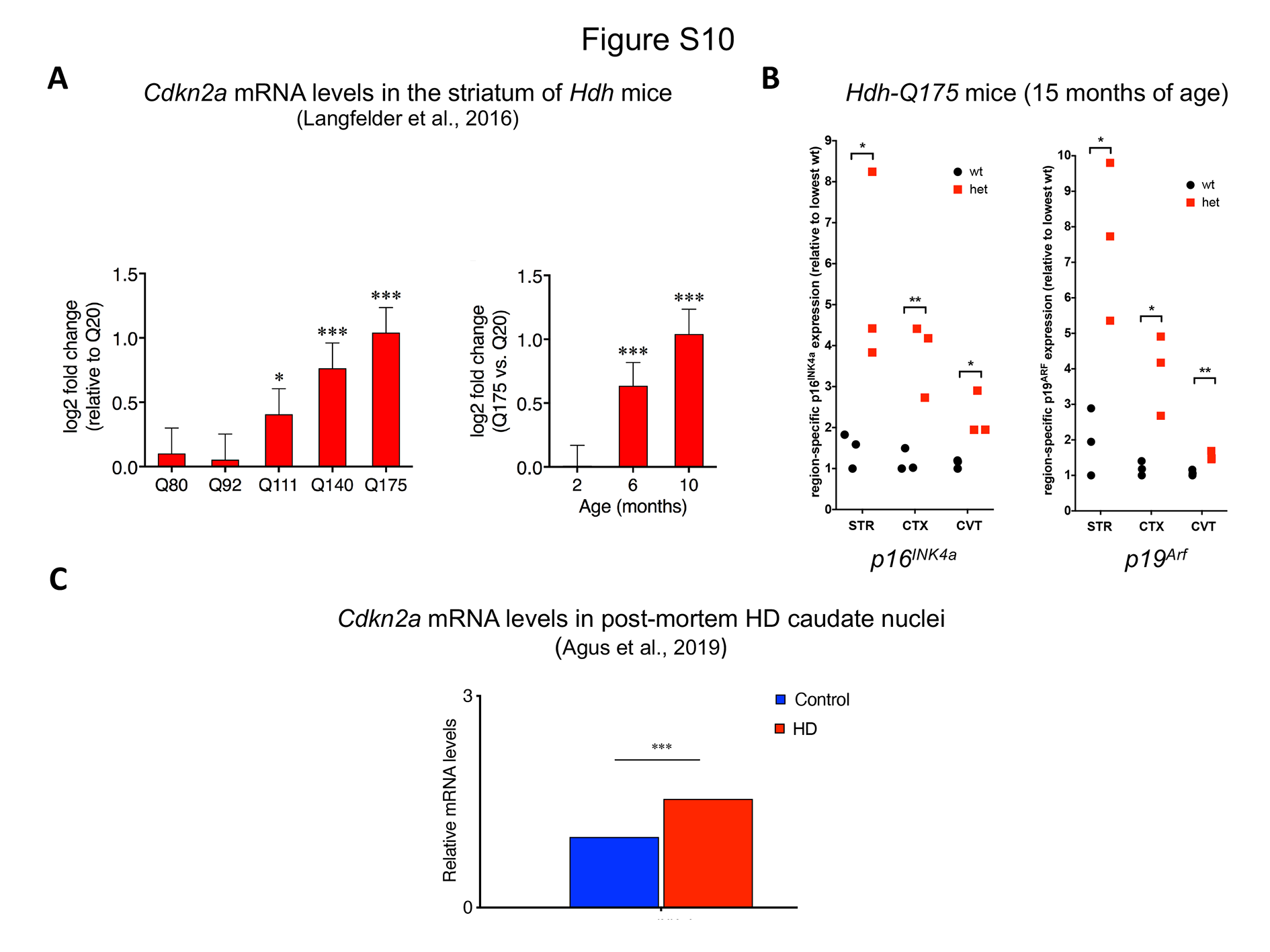

Supplement: Supplementary file 10 [file ACEL-19-e13226-s010.tif]
