# Supplementary material for: FOXO3 targets are reprogrammed as Huntington's disease neural cells and striatal neurons face senescence with p16INK4a increase
Source: Aging Cell. 2020 Nov 6;19(11):e13226. doi: 10.1111/acel.13226 (PMC7681055; doi:10.1111/acel.13226)
Supplement: Supplementary file 16 [file ACEL-19-e13226-s016.docx]

**Supporting information**

**Supporting methods**

**Protein co-immunoprecipitation assays**

HEK 293T cells were transfected with pcDNA3.1-Myc-Ryk-ICD or empty vector using JetPrime (PolyPlus Transfection, POL-114-07). 48 h after transfection, cells were treated with 50 µM H_2_O_2_ (AAT Bioquest, 11004) for 1 h 30 min then washed once with cold Dulbecco's phosphate buffered saline (DPBS, Life Technologies) and lysed in 25 mM Tris-HCl, pH 7.4, 150 mM NaCl, 5 mM EDTA, 1% Triton X-100, 10 mM sodium pyrophosphate, 10 mM b-glycerophosphate, 1 mM sodium orthovanadate, 10% glycerol, supplemented with protease inhibitors (Halt™ Protease Inhibitor Cocktail, ThermoFisher Scientific, 78430). Lysates were cleared for 10 min at 16,000 g at 4 °C. BCA assay (ThermoFisher Scientific, 23225) was performed to determine protein concentration.

The cell lysates were precleared with Dynabeads Protein G (Life Technologies, 10003D) for at least 1 h at 4 °C. Immunoprecipitations were carried out by incubating equal amounts (2 mg) of total lysate with either 5 μg of ß-catenin antibody (BD 610154) or 8 µg of FOXO3 antibody (Santa Cruz, H-144 sc-11351X) at 4°C overnight with gentle rotation. As a negative control we used normal mouse IgG, ThermoFisher Scientific MA110407 and normal rabbit IgG, Cell Signaling Technology 2729S, respectively). Immune complexes were captured by incubation with 25 μl of pre-washed Dynabeads Protein G (Life Technologies) for 4 h at 4 °C. Beads were washed 3 times for 10 min at 4 °C (with end-over-end rotation) in cold lysis buffer plus protease inhibitors. The complexes were eluted at 100° C for 10 min in 1x LDS buffer (Life Technologies, NP0007). Samples were resolved on NuPAGE 4–12% gels in 1X NuPAGE MES SDS buffer (Life Technologies, NP0335BOX and NP0002 respectively) and transferred to a PVDF membrane (Bio-Rad, 162-0177). Western blot analysis was performed under standard conditions using anti-FOXO3 (H-144, Santa Cruz, 1:1000), anti-Myc (Cell Signaling Technology, 2278S, 1:1000) and anti-ß-catenin (Cell Signaling Technology, 9582S, 1:1000).

**Deletion mapping assays**

To determine the binding site of the Ryk-ICD fragment on ß-catenin, we constructed deletion mutants of ß-catenin for the Armadillo repeat region. The full-length human ß-catenin with a C-terminal Flag tag in pcDNA3.1 (Plasmid #16828, Addgene) was used to generate deletion mutants using a PCR fusion-based approach. We generated constructs encoding ß-catenin mutants deleted for amino acids 277 to 488 (∆277-488) and 489 to 593 (∆489-593). We designed primers immediately upstream and downstream of the sequence to be deleted to carry short overlapping ends (22-24 bp). All mutants contained a C-terminal Flag tag. We used primers as follows: ß-catenin ∆277-488, Forward 5’-GGATCCCCAGCGTGGACAATGGCTACTC-3’ and Reverse 5’-cttaaccacaactggtagtccataagctaaacgcactgccatttt-3’ for amplifying the region encoding amino acids 1-276 and Forward 5’-tatggactaccagttgtggttaag-3’ and Reverse 5’-TCTAGATGCATGCTCGAGCGGCCGCTTACTTGTCATCGTCGTCCTTGTA-3’ for amplifying the region encoding amino acids 489-781; ß- ∆489-593, Forward 5’-GGATCCCCAGCGTGGACAATGGCTACTC-3’ and Reverse 5’- ctgcacaaacaatggaatggtattgtgaaggcgaactgcattctgggc-3’ for amplifying the region encoding amino acids 1-488 and Forward 5’- aataccattccattgtttgtgCAG-3’ and Reverse 5’-TCTAGATGCATGCTCGAGCGGCCGCTTACTTGTCATCGTCGTCCTTGTA-3’ for amplifying the region encoding amino acids 594-781. For each construct, the two PCR products were fused by nested PCR using primers 5’-GGATCCCCAGCGTGGACAATGGCTACTC-3’ and 5’-TCTAGATGCATGCTCGAGCGGCCGCTTACTTGTCATCGTCGTCCTTGTA-3’. BamHI and XbaI sites in sense and antisense primers respectively are underlined. The amplified fragments were cloned into the pGEM-T easy vector (Promega, A1360), sequenced and subcloned into the BamHI/XbaI sites (Invitrogen Inc.). All constructs were verified for sequence integrity.

HEK293T cells were transfected with Myc-Ryk-ICD and wild-type or mutant ß-catenin-Flag constructs ∆277-488 and ∆488-593 or insertless vector using JetPrime. 48 h after transfection, cells were treated with 50 µM H_2_O_2_ for 1 h 30 min, washed once with cold DPBS and with 25 mM Tris-HCl, pH 7.4, 150 mM NaCl, 5 mM EDTA, 1% Triton X-100, 10 mM sodium pyrophosphate, 10 mM b-glycerophosphate, 1 mM sodium orthovanadate, 10% glycerol, supplemented with protease inhibitors. Lysates were cleared for 10 min at 16,000 g at 4 °C. Protein quantification was performed by using BCA assays (ThermoFisher Scientific, 23225). Lysates were precleared with Dynabeads Protein G for at least 1 h at 4 °C. Immunoprecipitations were carried out by incubating equal amounts (2 mg) of cell lysates with 5 µg anti-FLAG (ThermoFisher Scientific, PA-1-984B) at 4 °C overnight with gentle rotation. As a negative control, we used an antibody of the same isotype and species (normal rabbit IgG, ThermoFisher Scientific, 10500C). Immune complexes were captured by incubation with 25 μl pre-washed dynabeads protein G (Life Technologies, 10003D) for 4 h at 4 °C. Beads were then washed three times for 10 min each at 4 °C (with end-over-end rotation) in cold lysis buffer containing protease inhibitors. Immunoprecipitated complexes were eluted at 100° C for 10 min in 1x LDS sample buffer. Samples were resolved on NuPAGE 4–12% gels in 1X NuPAGE MES SDS buffer and transferred to PVDF membranes. Western blot analysis was performed under standard conditions using the following antibodies: anti-Myc (ThermoFisher Scientific, MA1-980, 1:1000) and anti-FLAG (ThermoFisher Scientific, MA1-91878, 1:1000).

**Immunofluorescence analysis for quantification of FOXO3 levels**

NSCs were grown in Millicell® EZ 8-chamber Slides (EMD Millipore, PEZGS0816) coated with pO/L at 40000 cells/well. After 48 h, complete NPM was switched to medium without bFGF and LIF for 4 h followed by treatment with 20 µM LY294002 (Selleckchem, S1105) or vehicle for 1 h 30 min. Cells were fixed with 4% paraformaldehyde (ThermoFisher Scientific) for 20 min at room temperature (RT), washed twice with DPBS, permeabilized with 0.2% Triton X-100 (Sigma-Aldrich) in DPBS for 15 min at RT and washed twice with DPBS. Cells were blocked using 1% bovine serum albumin (BSA), 0.1% Triton 100-X in DPBS for 1 h at RT, then incubated overnight at 4° C with FOXO3 antibody (H-144, Santa Cruz, 1:200). Cells were washed with DPBS thrice, and incubated with goat-anti-rabbit Alexa Fluor 555 (Life Technologies, 1:200) in the dark for 2 h at RT. Following 3 washes with DPBS, coverslips were mounted using ProLong Gold antifade with DAPI (Life Technologies, P36941) and allowed to cure overnight at RT in the dark before imaging. Imaging was performed using an SP5 Leica Confocal Microscope (Leica Microsystems, Wetzlar, Germany), using the same settings for each condition tested in order to ensure data comparability. Images were analyzed using ImageJ software (<https://imagej.nih.gov/ij/>). First, DAPI images were used to create *a* binary mask to define regions of interest for analysis. Then, this mask was applied to the FOXO3 images to measure the fluorescence intensity in nuclei.

**Messenger RNA isolation and sequencing**

The RNA-seq samples corresponding to FOXO3 induction into the nucleus were prepared in duplicate. Cells were amplified in culture for a limited number of passages (7−12). Briefly, 15 millions HD (72Q/21Q) and Corrected C116 were transfected with validated siRNAs (Eurofins MWG Operon). Two Ryk siRNAs and scrambled RNAs were tested by qRT-PCR. The siRNAs were *Ryk* siRNA-1 5’-GCAAGUUAGUAGAGGCCAA-3’ and scrambled RNA-1 5’-AUCGAAGCUAGCGAUGAGA-3’ and *Ryk* siRNA-2 5’-AGUCUGGUUAAUAACGAGU-3’ and scrambled RNA-2 5’-GAAGGUUCGCGAUUAAUAU-3’. Cells were transfected by electroporation (Neon Transfection System 100µl kit, Life Technologies MK10096) according to the manufacturer’s instructions. Two days after transfection, complete NPM was switched to medium without bFGF and LIF for 4 h and cells were treated with 20 µM LY294002 for 1 h 30 min to activate endogenous FOXO3 as described (Brunet et al., 1999). Total RNA was isolated and purified using the RNeasy Mini kit (Qiagen, 74104) and DNase treated using the DNA-*free*^TM^ DNA removal kit (Life Technologies, AM1906) as per the manufacturer’s instructions. The mRNA libraries were prepared using standard protocols from Illumina and sequenced with the Illumina HiSeq Genome Analyzer for generating single-end 125 bp reads.

The RNA-seq samples corresponding to FOXO3 knockdown were prepared in triplicate. FOXO3 siRNAs (ON-TARGET plus SMART pool, L-003007-00-0020) and negative control siRNAs (ON-TARGET plus Non-targeting Control pool, D-001810-10-20) were obtained from Dharmacon (GE-Healthcare). Human NSCs were transfected using the Neon System 100 μl kit (Life Technologies MK10096) according to the manufacturer’s guidelines. Briefly, cells were harvested with Stempro Accutase (Life Technologies, A1110501), washed with DPBS and resuspended in Buffer R at 2 x 10^7^ cells/ml. 2 x 10^6^ cells were mixed with 250 nM siRNA. Conditions used for the electroporation were pulse voltage 1400 V, pulse width 20 ms and 2 pulses. Cells were seeded in 6-well matrigel-coated plates with 2 ml pre-warmed growth medium without antibiotics, and incubated at 37 °C. 48 h after transfection, complete NPM was replaced with medium without bFGF and LIF for 6 h before total RNA extraction. Total RNA was isolated and purified using the NucleonSpin RNA kit (Machery-Nagel 740955.50) and DNase treated using the DNA-*free*^TM^ DNA removal kit (Life Technologies, AM1906) as per the manufacturer’s instructions. The mRNA libraries were prepared using standard protocols from Illumina and sequenced with the Illumina NextSeq 500 for generating single-end 150 bp reads.

In both case, preparation and sequencing of mRNA libraries was performed by FASTERIS SA (Switzerland).

**Chromatin immunoprecipitation and sequencing**

HD and C116 NSCs were amplified in culture for a limited number of passages (7−12), and ChIP experiments were performed using the Agilent Mammalian ChIP-on-chip protocol (Agilent Technologies). For performing FOXO3 ChIP-seq, 80-100 million cells were transfected with Ryk siRNA and scrambled RNA (using electroporation as described above) and used to generate Illumina single-end libraries, the latter step performed by Fasteris SA (Switzerland). Two days after transfection, we switched complete NPM to medium without bFGF and LIF for 4 h and treated cells with 20 μM LY294002 for 1 h 30 min to activate endogenous FOXO3 as described (Brunet et al., 1999). Cells were cross-linked for 10 min with 1% PFA. Cross-linking was stopped by addition of 0.125 M glycine, followed by incubation for 5 min at room temperature. Cells were washed with cold DPBS, lysed in Lysis Buffer 1 (LB1) and rocked for 10 min at 4 °C. Cell lysates were cleared at 1,350 g for 5 min at 4°C and pellets were resuspended in Lysis Buffer 2 (LB2) and rocked for 10 min at 4 °C. Pellet nuclei were resuspended in Lysis Buffer 3 (LB3) and rocked 10 min at 4° C. Chromatin was sheared by sonication using a Bioruptor Plus device (Diagenode) at 20 times for 30 sec ON–30 sec OFF and centrifuged at 16,000 g for 15 min at 4 °C. For immunoprecipitation, we used 5 μg of a ChIP grade FOXO3 ‘NFL’ antibody (Brunet et al., 1999) or IgG antibdody (Cell Signaling, 2729) coupled to Dynabeads Protein G (Life Technologies). Chromatin was incubated overnight with antibody-coupled beads. Beads were washed with RIPA buffer 3 times and once with Tris-EDTA plus 50 nM NaCl. Chromatin was eluted in Elution Buffer at 65° C for 15 min, then reversed cross-linked by incubating at 65 °C overnight. DNA was extracted using phenol/chloroform and purified using a PCR purification kit (Qiagen, 28104). ChIP-seq libraries were prepared for sequencing using standard Illumina protocols as performed by Fasteris SA, Switzerland. DNA sequencing was carried out at Fasteris using a Illumina HiSeq Genome Analyzer sequencer.

**Next-generation sequencing data analysis**

Next generation sequencing data analyses were performed using the Galaxy framework developed by the ARTbio bioinformatics platform at the Institute of Biology Paris-Seine (<https://mississippi.snv.jussieu.fr/>).

**RNA-seq data analysis**

The raw sequencing reads corresponding to FOXO3 induction into the nucleus were pre-processed in order to discard both adapter sequences and low quality reads using the Trimmomatic and FASTQ Groomer tools. The filtered reads were then mapped to the human reference genome hg38 (UCSC 2013 release) by using Bowtie2 (Langmead & Salzberg, 2012). Duplicate and unmapped reads were removed using SAM Tools (Li et al., 2009). Read distributions were calculated using the featureCounts tool (Liao, Smyth, & Shi, 2014). To test for differentially expressed genes, reads were analyzed using the R/Bioconductor package edgeR (Robinson, McCarthy, & Smyth, 2010). For genes with very low read counts, a prior filtering step was performed to keep those with at least ten counts in all samples. Differential expression analysis was assessed using the exact negative binomial test and a false discovery rate (FDR) correction of 5%. The raw read counts from the RNA-seq data was first filtered to remove genes with a read count less than 5 in all samples. Principal component analysis (PCA) was performed upon normalizing raw RNA-seq readcounts using DESeq2 (1.22.2). PCA analysis was then performed using the R function prcomp (3.5.1). A 3D plot was generated using the R package rgl (0.100.30) to visualize the first 3 principal components. The volcano plots were generated using the R package EnhancedVolcano (1.0.1). The log fold change values obtained after the differential expression analysis between FOXO3 nuclear induction vs control were used as the input data. The data are available at GSE109873, subseries GSE109872.

The raw sequencing reads corresponding to FOXO3 knockdown were pre-processed in order to perform Quality control of the reads using fastqc and discard adapter sequences using Clip adapter (Galaxy Version 2.2.0). The reads were then mapped to the human reference genome hg38 (UCSC 2013 release) by using HISAT2 (Kim, Langmead, & Salzberg, 2015). Read distributions were calculated using the featureCounts tool [62]. To test for differentially expressed genes, reads were analyzed using the R/Bioconductor package DESeq2 version 1.18.1 (Love, Huber, & Anders, 2014). For genes with very low read counts, a prior filtering step was performed to keep those with a sum of at least ten counts in all samples. An adjusted p-value was calculated and a false discovery rate (FDR) of 1% was applied.  Analysis of FOXO3 dependence for gene expression in basal compared to stressed conditions was performed using the R function pnorm. The data are available at GSE109873, subseries GSE109869.

**ChIP-seq analysis/Processing of ChIP-seq reads**

Reads of 50 bp lengths were generated using and Illumina HiSeq Genome analyzer. Raw sequencing reads were mapped to the human reference genome (hg38 2013 release) using Bowtie2 (Langmead & Salzberg, 2012). Duplicate and unmapped reads were removed using SAM tools. Peaks were called using MACS2 (Zhang et al., 2008) set to default parameters except for read lengths set at 100 and cut-offs on *P* value set to 0.05. Deduction of input (whole cell extract) and mock antibody signal was carried out using bdgcmp available from MACS2. Peaks corresponding to induction of FOXO3 in the nucleus were then called using the tool bdgpeakcall available from MACS2. Generated peaks were annotated using the R/Bioconductor package ChIPseeker (Yu, Wang, & He, 2015) and a peak calling set to ±5 kb from a transcription start site (TSS). DeepTools (Ramirez et al., 2016) were used to perform binding enrichment across TSSs with sorting based on mean and k-means clustering.

**Integrative analysis of RNA-seq and ChIP-seq data**

Comparison of RNA-seq and ChIP-seq data was performed using Monte Carlo simulations with 10,000 repetitions for testing all overlaps between the 4 experimental conditions. In addition, global and pairwise comparisons of ChIP-seq signals (or peak score distributions) for the 4 conditions were examined using the Chi-squared test in R.

**Motif discovery and enrichment analysis**

Analysis of sequence motifs was performed using tools of the MEME Suite (Stuge & Ellingsen, 1991) for the MACS2 peaks corresponding to the promoter regions within ±250 bp. Default parameters were used except for MEME Motif Count (value: 8), DREME Motif E-value (value: 0.5) and CentriMo Match Score (value: 8). The significance of detected motifs was assessed using an E-value <0.5. Motif annotation was then performed using the HOmo sapiens COmprehensive MOdel COllection database (HOCOMOCO v10) (Kulakovskiy et al., 2013). To determine the family-wide significance of motifs (*e.g.* the FOXO family), the top p-value was considered for each family.

**Orthology analysis**

The human-mouse orthologous gene pairs were extracted from Ensembl database with the DIOPT tool (https://www.flyrnai.org/cgi-bin/DRSC_orthologs.pl) with parameters set to best orthologs of mouse genes. Fischer’s exact test were used to test for statistical significance of overlaps.

**Biological content analysis**

Biological content analysis was using EnrichR (http://amp.pharm.mssm.edu/Enrichr/). Additionally, networks containing subgroups of FOXO3 direct targets as seed genes and their level-1 neighbors (for a total number of neighbors set at 60-200) were derived from the high coverage and probabilistic functional network STRING v10.0 (Szklarczyk et al., 2015) and displayed using confidence view, selectively performed using high-confidence (STRING probability score ≥ 0.7) edges based on information from databases and experiments. Networks were represented using Cytoscape (http://www.cytoscape.org/). Networks were annotated with signaling pathway and biological process information as provided in STRING. Seed genes (F3T-INs) in these networks were annotated further with information on the magnitude of regulation by FOXO3, as inferred from log2 fold changes in RNA-seq data, information from FOXO3 knockdown data and with information on gene deregulation in HD compared to C116 NSCs (Ring et al., 2015). To prioritize F3Ts for validation studies based on the predicted impact of F3T reprogramming on signaling-pathway and cellular activity, we selected the short paths interconnecting at least three categories of F3T (lost or gained, positively or negatively regulated by FOXO3). The magnitude of regulation by FOXO3 and novelty compared to the literature were also used for prioritizing F3Ts.

**Transfection of human NSCs**

FOXO3 siRNAs (ON-TARGET plus SMART pool, L-003007-00-0020), ETS1 siRNAs (ON-TARGET plus SMART pool, L-003887-00-0005), ETS2 siRNAs (ON-TARGET plus SMART pool, L-003888-00-0005) and negative control siRNAs (ON-TARGET plus Non-targeting Control pool, D-001810-10-20) were obtained from Dharmacon (GE-Healthcare). Previously validated siRNAs targeting exon 1 of CDKN2A were p16^INK4A^ siRNA-1 (5’-AACGCACCGAATAGTTACGGT-3’) (Bond et al., 2004) and p16^INK4A^ siRNA-2 (5'-CUGCCCAACGCACCGAAUA-3') (Kan et al., 2012) and non-specific control 47% CG siRNA were obtained from Eurofins Genomics. Human NSCs were transfected using the Neon System 100 μl kit (Life Technologies MK10096) according to the manufacturer’s guidelines. Briefly, cells were harvested with Stempro Accutase (Life Technologies, A1110501), washed with DPBS and resuspended in Buffer R at 2 x 10^7^ cells/ml. 2 x 10^6^ cells were mixed with 250 nM siRNA. Conditions used for the electroporation were pulse voltage 1400 V, pulse width 20 ms and 2 pulses. Cells were seeded in 6- well matrigel-coated plates with 2 ml pre-warmed growth medium without antibiotics, and incubated at 37 °C. 48 h after transfection, complete NPM was replaced with medium without bFGF and LIF for 6 h before total RNA extraction.

Human NSCs were transfected with cDNAs using jetOptimus reagent (PolyPlus Transfection) according to the manufacturer’s guidelines within the recommended reagent/DNA ratio range. Briefly, cells were plated at density 5x10^6^ cells/well in 12 well-plates. Twenty-four hours after seeding, complete NPM in all wells was exchanged for 1 ml of fresh medium following transfection with the 1µg pcDNA3.1-FLAG-FOXO3 WT (Addgene Plasmid #8360**)**, pcDNA3.1-FLAG-FOXO3-TM (Addgene Plasmid #1070**9)** or pcDNA3.1. 48 hours after transfection, complete NPM was replaced with medium without bFGF and LIF for 9 h before total RNA extraction. Cell viability was detected 48h after transfection using the CellTiter-Glo® Luminescent Cell Viability Assay (Promega, G7571) according to the manufacturer’s protocol. Briefly, a volume of CellTiter-Glo Reagent equal to the volume of culture medium was added to each well, and mixed for 2 minutes on orbital shaker. The plate was then incubated at RT for 10 min and luminescence of each sample measured using the plate-reader FLUOstar Optima (BMG Labtech).

**Gene expression analysis**

Total RNA was isolated from cells using the RNeasy kit (Qiagen), and DNase treated using the DNA-free DNA removal kit according to the manufacturer’s instructions Kit (Ambion). Equal amounts of total RNA (1 μg) was reverse-transcribed using the RevertAID First Strand cDNA synthesis kit (Thermo Fisher scientific, K1622), according to the manufacturer’s instructions. The first strand cDNA was diluted and used as template in the real-time quantitative-PCR analysis. The LightCycler 480 Real-Time PCR System was used to perform the qRT-PCR using GoTaq qPCR Master Mix (Promega, A6002). qRT PCR experiments were performed in triplicate using the following primers: *FOXO3*: Forward: 5’-AGGGAGTTTGGTCAATCAGAA-3’, Reverse: 5’- TGGAGATGAGGAATCAAAGTT-3’; Ryk: Forward: 5’-CCACTTCTACGCGTGTGTTT-3’, Reverse: 5’- GCCCTTGGGAACTACTGC-3’; *p16^INK4^*: Forward: 5’-CCAACGCACCGAATAGTTACG-3’, Reverse: 5’- GCGCTGCCCATCATCATG-3’; *p14^ARF^*: Forward: 5‘-CCCTCGTGCTGATGCTACTG-3’, Reverse: 5’-CATCATGACCTGGTCTTCTAGGAA-3’; *CDKN2AIP*: Forward: 5’-GTGTATAGGGTCGGCCATCAA-3’, Reverse: 5’-CCTGCCGTTGTTACCTGAGAG-3’; *SERTAD1*: Forward: 5’- CTCAAGCTCCACCACAGCCT-3’, Reverse: 5’-AGTGTTCACGACCAGCACCA-3’; *ETS2*: Forward: 5’-CTGGGCATTCCAAAGAACCC-3’, Reverse: 5’-CCAGACTGAACTCATTGGTGG-3’; *ETS1* Forward: 5’-GGGAGGACCAGTCGTGGTAAA-3’, Reverse: 5’-CACGCTGCAGGCTGTTGAAAG-3’; *p21^CIP1^*: Forward: 5’-CACCGAGGCACTCAGAGGAG-3’, Reverse 5’-CCGCCATTAGCGCATCACAG-3’; *p27^KIP1^*: Forward: 5’-TAATTGGGGCTCCGGCTAACT-3’, Reverse: 5’-TGCAGGTCGCTTCCTTATTCC-3’; *HRPT*: Forward: 5’-ATGCTGAGGATTTGGAAAGG-3’ Reverse: 5’-CTCCCATCTCCTTCATCACA-3’; *ACTB*: Forward: 5‘-CCAACCGCGAGAAGATGA -3’, Reverse: 5’-CCAGAGGCGTACAGGGATAG-3’. QRT-PCR was performed at 95 °C for 10 min, followed by 40 cycles at 95 °C for 15 sec, 60 °C for 30 sec, and 72 °C for 30 sec. Data were analyzed using the LightCycler 480 software (Roche) and advanced relative quantification method. Gene expression was quantified by the mean cycle threshold (Ct) value for triplicate measurements. Target gene expression was normalized to two housekeeping genes (HPRT and ACTB) according to the 2-ΔΔCt formula. Statistical analyses (2-way ANOVA and t-tests) were performed using GraphPad Prism v6.

For biochemical analysis of Activin A and MSNs total RNA was isolated from NSCs and MSNs using ISOLATE II RNA Mini Kit (Bioline). cDNA was prepared from 1 μg of RNA in a total reaction volume of 20 μl using the SensiFAST cDNA synthesis kit (Bioline). RT-PCR reactions were setup in a 384-well format using 2X SensiFAST Probe No-ROX kit (Bioline) and 1 μl cDNA per reaction in a total volume of 10 μl. RT-PCR was performed on the Roche LightCycler 480 instrument. For quantification, the threshold cycle, Ct, of each amplification was determined by using the second derivative maximum method. The 2^–^**^ΔΔ^**^Ct^ method was used to determine the relative expression levels of each gene normalized against the housekeeping gene b-actin. The primers used were as follows: *p16^INK4a^*: Forward: 5’-CAGCAGCATGGAGCCTTC-3’, Reverse: 5’-CGTAACTATTCGGTGCGTTG-3’, Probe 67 and Forward: 5’-CTGCCCAACGCACCGAATA-3’, Reverse: 5’-GCTGCCCATCATCATGACCT-3’, Probe FAM; FOXO3: Forward: 5’-CTTCAAGGATAAGGGCGACA-3’, Reverse: 5’-CGACTATGCAGTGACAGGTTG-3’, Probe 11; MMP3: Forward 5’-GCTGATATAATGATCTCTTTTGCAGT-3’, Reverse: 5’-CATAGGCATGGGCCAAAA-3’, Probe 85.

Additonal primers for senescent markers are shown below along with Probe number are below.

|  |  |  |
| --- | --- | --- |
| CDKN2AIP-F-41 | 41 | gcgaaccacgtcttcctc |
| CDKN2AIP-R-41 | 41 | ttggagcatctgtcactttga |
| ETS1-F-UPL69 | 69 | aagtcctggaagggagatcg |
| ETS1-R-UPL69 | 69 | gcatacagcttttattccaagtca |
| SELL-F-UPL72 | 72 | agttgtgggggtggacaat |
| SELL-R-UPL72 | 72 | cagcagtcggttccatgat |
| IGFBP7-F-UPL67 | 67 | actggctgggtgctggta |
| IGFBP7-R-UPL67 | 67 | tggatgcatggcactcata |

For analysis of mRNA levels in brain samples from *Hdh*-Q175 mice, 3 male mice of each genotype (heterozygous and corresponding wildtype control) were sacrificed at 15 months of age by cervical elongation. Brains were immediately dissected and the striatum, cortex and cerebelum of these mice were snap frozen in liquid nitrogen. RNA was extracted using the Nucleospin kit from Macherey-Nagel, following manufacturer’s instructions. RNA samples were then incubated with DNAse (DNA-free DNA removal kit, ThermoFischer Scientific) to avoid genomic DNA contamination. Reverse transcription (RevertAid First Strand Synthesis kit, ThermoFischer Scientific) and qPCR (GoTaq qPCR Master Mix, Promega) was performed using manufacturer’s instructions on a Roche LC480 instrument. Primer sequences are: p16^INK4a^ *forward* 5’-AGGGCCGTGTGCATGACGTG-3’, *reverse* 5’-GCACCGGGCGGGAGAAGGTA-3’; p19^ARF^ *forward* 5’-CATGTTGTTGAGGCTAGAGAGG-3’, *reverse* 5’-TCGAATCTGCACCGTAGTTG-3’; HPRT *forward* 5’-ATTATGCCGAGGATTTGGAA-3’, *reverse* 5’-CCCATCTCCTTCATGACATCT-3’.

**Immunofluorescence analysis and quantification of p16^INK4a^ and HMGB1**

NSCs plated (and differentiated into MSNs) in 8-well Nunc Lab-Tek II Chamber Slides (Thermo Fisher Scientific) were fixed with 4% paraformaldehyde for 15 min at room temperature (RT), and washed twice with PBS. Cells were permeabilized with 0.25% Triton X-100 (Sigma-Aldrich) in PBS for 15 min at RT, then washed twice with PBS. Blocking was performed using 5% donkey serum and 1% BSA in PBS for 30 min at RT. Cells were washed with PBS, and incubated overnight at 4 °C with primary antibody, washed with PBS three times, and incubated with fluorescent secondary antibody in the dark for 2 h at RT. Following three washes with PBS, coverslips were mounted using ProLong Gold antifade with DAPI (Thermo Fisher Scientific). Slides were cured for 24 h in the dark at RT, and imaging performed on Nikon Eclipse Ti-U microscope using the Plan Apo λ 20X/0.75 objective. Primary antibodies – p16^INK4a^ (Abcam, ab108349), HMGB1 (Abcam, ab18256), and Nestin (SCBT, sc-23927) – were used at a dilution of 1:100. Secondary Alexa Fluor antibodies were purchased from Invitrogen. Image analysis was performed using the Gen5 software. TIFF images were converted to monochrome images and single cell analysis was performed using DAPI-stained nuclei to define the region of interest. The HMGB1 ICC analysis for lentivirus transduced MSNs was carried out using a Biotek Cytation 4, Gen5 Image Software using advanced features in which the GFP positive cells were masked for the nuclear staining and the cytoplasmic signal was quantified.  MSNs were transduced with lentivirus for 96 hours and then fixed as described above.  HMGB1, Rbt, Abcam AB18256​ at 1:100 for ICC as described above.

**Senescence-associated ß-galactosidase (SA-ß-gal) staining**

NSCs were cultured as described above with the addition of 25 ng/ml Activin A (Peprotech, AF-120-14E). NSCs were stained using the Senescence staining kit (#9860, Cell Signaling Technology). Nuclei were stained with DAPI, and coverslips were mounted as described above. Images were captured using the Lionheart FX Automated Microscope and a 10X Plan Fluorite WD 10 NA 0.3 objective. Image analysis was performed using the Gen5 software. TIFF images were converted to monochrome images and single cell analysis was performed using DAPI-stained nuclei to define the region of interest and average SA-ß-gal intensity/cell was quantified.

**Cell proliferation assays**

Human NSCs were seeded on 24-well plates at 0.5-1 x 10^5^ cells per well, 6 wells for per genotype. After 1, 2, 3, 4 and 5 days at 37 °C and 5% CO_2_, the medium was replaced with 500 µl fresh medium containing 10% v/v AlamarBlue® reagent (ThermoFisher Scientific, DAL1025) according to the manufacturer’s protocol. The plates were then incubated at 37° C for 3 hours. 100 µl from each well was transferred to a 96-well plate for reading. Fluorescence (excitation and emission wavelength 550 and 595 nm) was measured using the Infinite®F500 microplate reader (Tecan Genios). The 100% reduced form of AlamarBlue®, (*i.e.*, medium containing 10% v/v AlamarBlue® autoclaved at 121°C for 15 min) were used as positive control. Wells without cells with culture medium containing 10% v/v AlamarBlue were used as negative controls. The relative fluorescence intensity for each genotype and each day was calculated as the AlamarBlue® fluorescence signal of the sample at day X minus the signal of the negative control. Statistical analyses (2-way ANOVA) were performed using Prism v6.

**Cellular vulnerability assays**

Human NSCs were subjected to 24 h of growth factor deprivation as performed 48 h after cell transfection (by electroporation, as described above). Cell viability and caspase-3/7 activity were then detected using the ApoLive-Glo Multiplex Assay (Promega, G6410). Briefly, 10 µl of reagent (GF-AFC substrate) were added to each well, and gently mixed for 30 seconds. After incubation for 30 min at 37° C, fluorescence was measured using the plate-reader FLUOstar Optima (Ex at 360 nm, Em at 490 nm, BMG Labtech). Then, 50 μl of Caspase-Glo^®^ 3/7 reagent was added to each well, and gently mixed for 30 seconds. These plates were then incubated at RT for 30 min and luminescence of each sample measured using the plate-reader FLUOstar Optima (BMG Labtech). Caspase-3/7 assays were performed using 5 replicates/point and data expressed as Caspase-3/7 activity (RLU) divided by cell viability (RFU). Statistical analyses (paired t test) were performed using GraphPad Prism v6.

**Ethical statement**

In France, animals were handled and all experimental procedures were carried out in accordance with the guidelines of the French Agriculture and Forestry Ministry (decree 87849) and of the European Communities Council Directive (86/609/EEC).

**References in the supporting methods**

Brunet, A., Bonni, A., Zigmond, M. J., Lin, M. Z., Juo, P., Hu, L. S., . . . Greenberg, M. E. (1999). Akt promotes cell survival by phosphorylating and inhibiting a Forkhead transcription factor. *Cell, 96*(6), 857-868.

Eijkelenboom, A., Mokry, M., Smits, L. M., Nieuwenhuis, E. E., & Burgering, B. M. (2013). FOXO3 selectively amplifies enhancer activity to establish target gene regulation. *Cell Rep, 5*(6), 1664-1678. doi:10.1016/j.celrep.2013.11.031

Kim, D., Langmead, B., & Salzberg, S. L. (2015). HISAT: a fast spliced aligner with low memory requirements. *Nat Methods, 12*(4), 357-360. doi:10.1038/nmeth.3317

Kulakovskiy, I. V., Medvedeva, Y. A., Schaefer, U., Kasianov, A. S., Vorontsov, I. E., Bajic, V. B., & Makeev, V. J. (2013). HOCOMOCO: a comprehensive collection of human transcription factor binding sites models. *Nucleic Acids Res, 41*(Database issue), D195-202. doi:10.1093/nar/gks1089

Langmead, B., & Salzberg, S. L. (2012). Fast gapped-read alignment with Bowtie 2. *Nat Methods, 9*(4), 357-359. doi:10.1038/nmeth.1923

Lejeune, F. X., Mesrob, L., Parmentier, F., Bicep, C., Vazquez-Manrique, R. P., Parker, J. A., . . . Neri, C. (2012). Large-scale functional RNAi screen in C. elegans identifies genes that regulate the dysfunction of mutant polyglutamine neurons. *BMC Genomics, 13*, 91. doi:1471-2164-13-91 [pii]10.1186/1471-2164-13-91

Li, H., Handsaker, B., Wysoker, A., Fennell, T., Ruan, J., Homer, N., . . . Genome Project Data Processing, S. (2009). The Sequence Alignment/Map format and SAMtools. *Bioinformatics, 25*(16), 2078-2079. doi:10.1093/bioinformatics/btp352

Liao, Y., Smyth, G. K., & Shi, W. (2014). featureCounts: an efficient general purpose program for assigning sequence reads to genomic features. *Bioinformatics, 30*(7), 923-930. doi:10.1093/bioinformatics/btt656

Love, M. I., Huber, W., & Anders, S. (2014). Moderated estimation of fold change and dispersion for RNA-seq data with DESeq2. *Genome Biol, 15*(12), 550. doi:10.1186/s13059-014-0550-8

Miller, J. P., Yates, B. E., Al-Ramahi, I., Berman, A. E., Sanhueza, M., Kim, E., . . . Hughes, R. E. (2012). A genome-scale RNA-interference screen identifies RRAS signaling as a pathologic feature of Huntington's disease. *PLoS Genet, 8*(11), e1003042. doi:10.1371/journal.pgen.1003042PGENETICS-D-12-00404 [pii]

Paik, J. H., Ding, Z., Narurkar, R., Ramkissoon, S., Muller, F., Kamoun, W. S., . . . DePinho, R. A. (2009). FoxOs cooperatively regulate diverse pathways governing neural stem cell homeostasis. *Cell Stem Cell, 5*(5), 540-553. doi:S1934-5909(09)00509-8 [pii]10.1016/j.stem.2009.09.013

Ramirez, F., Ryan, D. P., Gruning, B., Bhardwaj, V., Kilpert, F., Richter, A. S., . . . Manke, T. (2016). deepTools2: a next generation web server for deep-sequencing data analysis. *Nucleic Acids Res, 44*(W1), W160-165. doi:10.1093/nar/gkw257

Renault, V. M., Rafalski, V. A., Morgan, A. A., Salih, D. A., Brett, J. O., Webb, A. E., . . . Brunet, A. (2009). FoxO3 regulates neural stem cell homeostasis. *Cell Stem Cell, 5*(5), 527-539. doi:S1934-5909(09)00510-4 [pii]10.1016/j.stem.2009.09.014

Ring, K. L., An, M. C., Zhang, N., O'Brien, R. N., Ramos, E. M., Gao, F., . . . Ellerby, L. M. (2015). Genomic Analysis Reveals Disruption of Striatal Neuronal Development and Therapeutic Targets in Human Huntington's Disease Neural Stem Cells. *Stem Cell Reports, 5*(6), 1023-1038. doi:10.1016/j.stemcr.2015.11.005

Robinson, M. D., McCarthy, D. J., & Smyth, G. K. (2010). edgeR: a Bioconductor package for differential expression analysis of digital gene expression data. *Bioinformatics, 26*(1), 139-140. doi:10.1093/bioinformatics/btp616

Stuge, U., & Ellingsen, J. E. (1991). [Frialit ceramic implants--a four-year follow-up study]. *Nor Tannlaegeforen Tid, 101*(9), 296-303.

Szklarczyk, D., Franceschini, A., Wyder, S., Forslund, K., Heller, D., Huerta-Cepas, J., . . . von Mering, C. (2015). STRING v10: protein-protein interaction networks, integrated over the tree of life. *Nucleic Acids Res, 43*(Database issue), D447-452. doi:gku1003 [pii]10.1093/nar/gku1003

Webb, A. E., Kundaje, A., & Brunet, A. (2016). Characterization of the direct targets of FOXO transcription factors throughout evolution. *Aging Cell, 15*(4), 673-685. doi:10.1111/acel.12479

Webb, A. E., Pollina, E. A., Vierbuchen, T., Urban, N., Ucar, D., Leeman, D. S., . . . Brunet, A. (2013). FOXO3 Shares Common Targets with ASCL1 Genome-wide and Inhibits ASCL1-Dependent Neurogenesis. *Cell Rep, 4*(3), 477-491. doi:S2211-1247(13)00327-6 [pii]10.1016/j.celrep.2013.06.035

Yu, G., Wang, L. G., & He, Q. Y. (2015). ChIPseeker: an R/Bioconductor package for ChIP peak annotation, comparison and visualization. *Bioinformatics, 31*(14), 2382-2383. doi:10.1093/bioinformatics/btv145

Zhang, Y., Liu, T., Meyer, C. A., Eeckhoute, J., Johnson, D. S., Bernstein, B. E., . . . Liu, X. S. (2008). Model-based analysis of ChIP-Seq (MACS). *Genome Biol, 9*(9), R137. doi:10.1186/gb-2008-9-9-r137

**Supporting tables**

**Table S1.** Definition of F3Ts in human C116 and HD NSCs. Sheet 1 shows the complete list of human genes that are differentially expressed upon FOXO3 induction into the nucleus (IN) compared to no FOXO3 induction (F3T-IN). This table is annotated with information on FOXO3 binding at promoters (-5 kb/+2 kb) and enhancers (± 20 kb outside the promoter regions), deregulation in HD NSCs, the same as the ones used herein, as previously reported (Ring et al., 2015), druggability, overlap with FOXO3 targets in other cell types as previously reported (Eijkelenboom, Mokry, Smits, Nieuwenhuis, & Burgering, 2013; Paik et al., 2009; Renault et al., 2009; Webb, Kundaje, & Brunet, 2016), and overlap with RNAi screens in a transgenic nematode (Lejeune et al., 2012) and human cell (Miller et al., 2012) models of HD pathogenesis. Sheet 2 shows the complete list of human genes that are differentially expressed upon cell stress (growth factor deprivation) in a FOXO3-knockdown (KD)-dependent manner. These genes are those for which the log fold change (LFC) of gene expression levels in stressed cells treated with FOXO3 siRNAs is no longer significant compared to the significant LFCs in stressed cells treated with non targeting control (NTC) pool of RNAs. These genes also comprise those for which there is a significant difference between log fold change (LFC) of gene expression levels in stressed cells treated with FOXO3 siRNAs compared to significant LFCs in stressed cells treated with NTC RNAs. LFCs were considered significant for a q-value < 0.1 (green cells) as determined using false discovery rate (FDR) analysis. Differences between LFCs were considered significant for a p-value < 0.05 as determined using the R function pnorm. A significant difference or a loss of LFC significance upon FOXO3 knockdown define the subgroup of F3T-IN-KD targets (blue cells). NA, not applicable.

**Table S2.** Table S1 extracts showing the list of F3T-INs that are gained in HD NSCs and their behavior upon silencing of Ryk (sheet 1), those that are lost in HD NSCs and their behavior upon reduction of Ryk expression (sheet 2) and those that are conserved in HD NSCs and their behavior upon reduction of Ryk expression (sheet 3).

**Table S3.** Comparison of FOXO3 binding sites in human C116 and mouse NSCs. See also Figure S3.

**Table S4.** F3T-IN targets reprogrammed (lost or gained) in human HD NSCs in a Ryk-independent manner (Table S1 extract). The sub-group of F3T-IN-KD targets is indicated by blue stars in Figure S4B.

**Table S5.** FOXO3 targets reprogrammed (lost or gained) in human HD NSCs in a Ryk-dependent manner (Table S1 extract). The sub-group of F3T-IN-KD targets is indicated by blue stars in Figure S4C.

**Supporting figures**

**
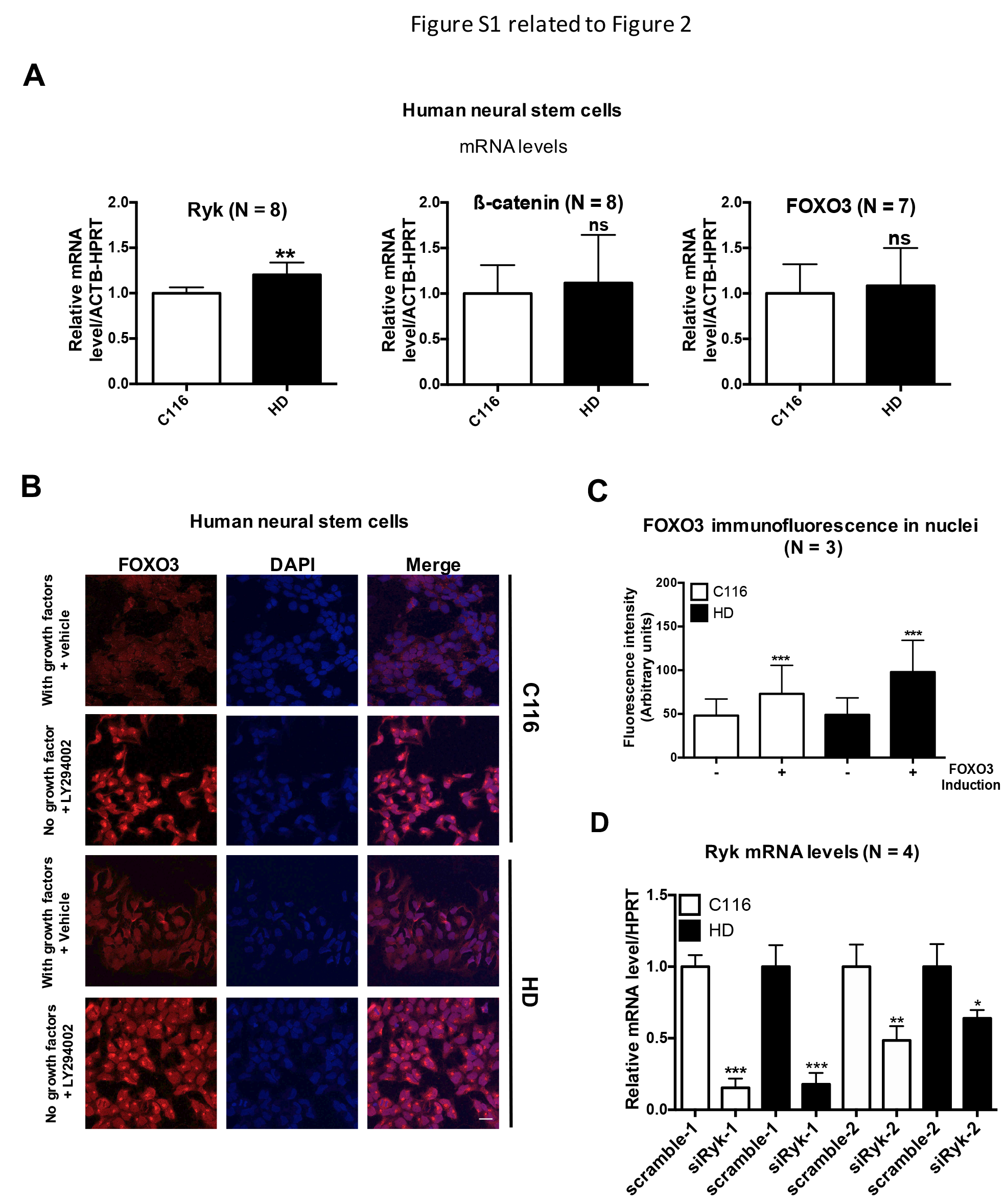
**

**FIGURE S1.** Gene expression analysis, FOXO3 induction and efficiency of Ryk silencing in HD and C116 NSCs. Related to Figure 2**.** (A) mRNA levels of Ryk, ß-catenin and *FOXO3* in HD (72Q/19Q) and C116 (21Q/19Q) neural stem cells as measured by using RT-PCR. Data are mean±SD. ***P* < 0.01 compared to C116 NSCs. (B) FOXO3 induction into the nucleus in HD and C116 NSCs. FOXO3 was induced in the nucleus by growth factor deprivation complemented by 20 μM LY294002 treatment for 90 minutes (see Methods). Representative confocal images of FOXO3 intracellular distribution upon induction into the nucleus of HD and C116 NSCs. (C) FOXO3 induction into the nucleus in HD and C116 NSCs. The nuclear localization of FOXO3 is increased in HD and C116 cells. Data are mean±SEM for the ratio Intensity/Area of FOXO3 signal in the nucleus (N = 3). ****P* < 0.001 compared to no induction. (D) Ryk mRNA levels are decreased by siRNA treatment in HD and C116 NSCs. Data are mean±SD (N = 3). ****P* < 0.001 and ***P* < 0.01 compared to scrambled RNA treatment.


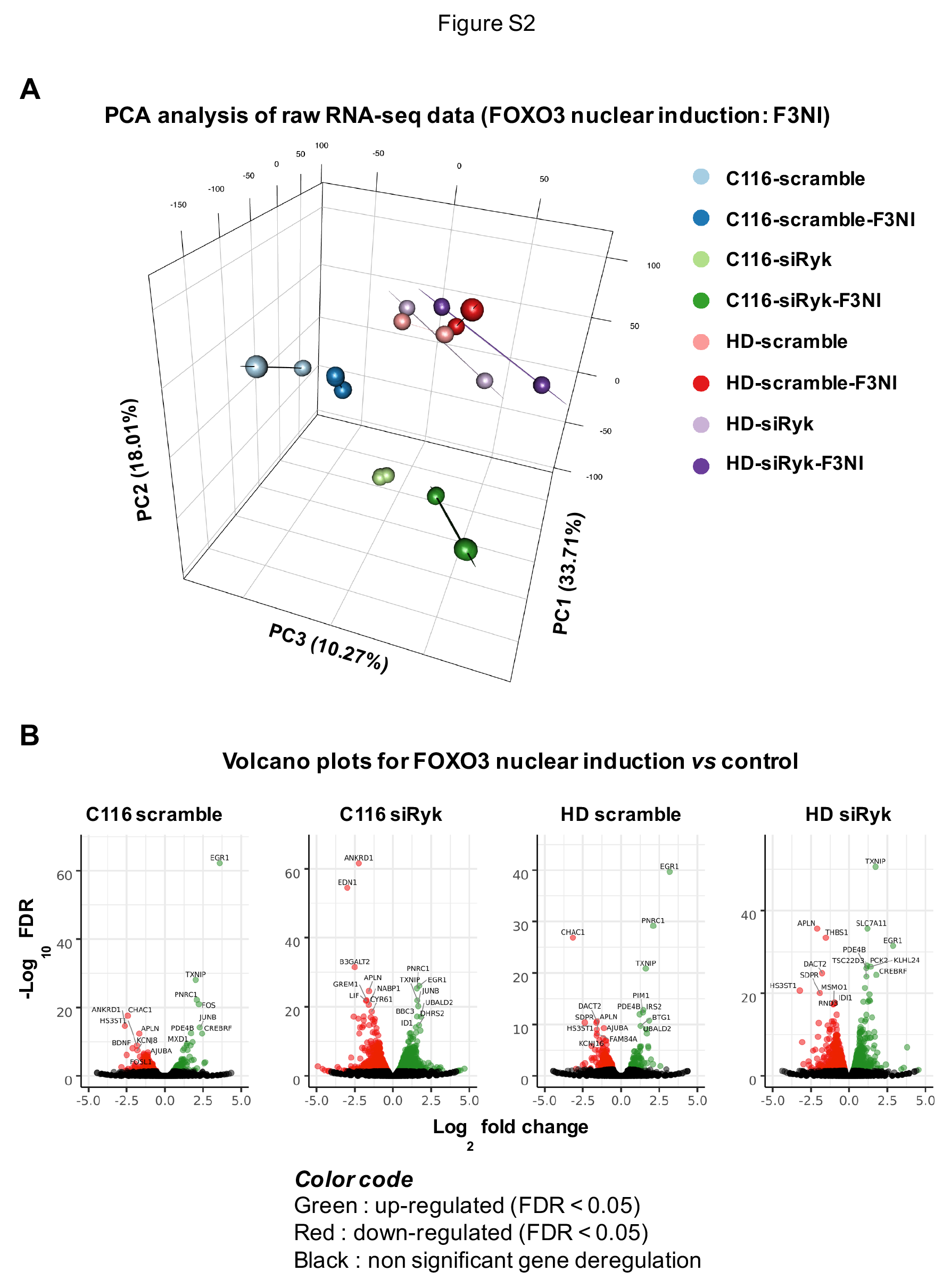


**FIGURE S2.** Overview of RNA-seq data upon FOXO3 nuclear induction in HD and C116 NSCs. (A) Principal component analysis of raw RNA-seq data. Each condition involves two technical replicates (almost indistinguishable) and two biological replicates. (B) The log fold change values obtained after the differential expression analysis between FOXO3 nuclear induction vs control were used to generate the volcano plots.

**
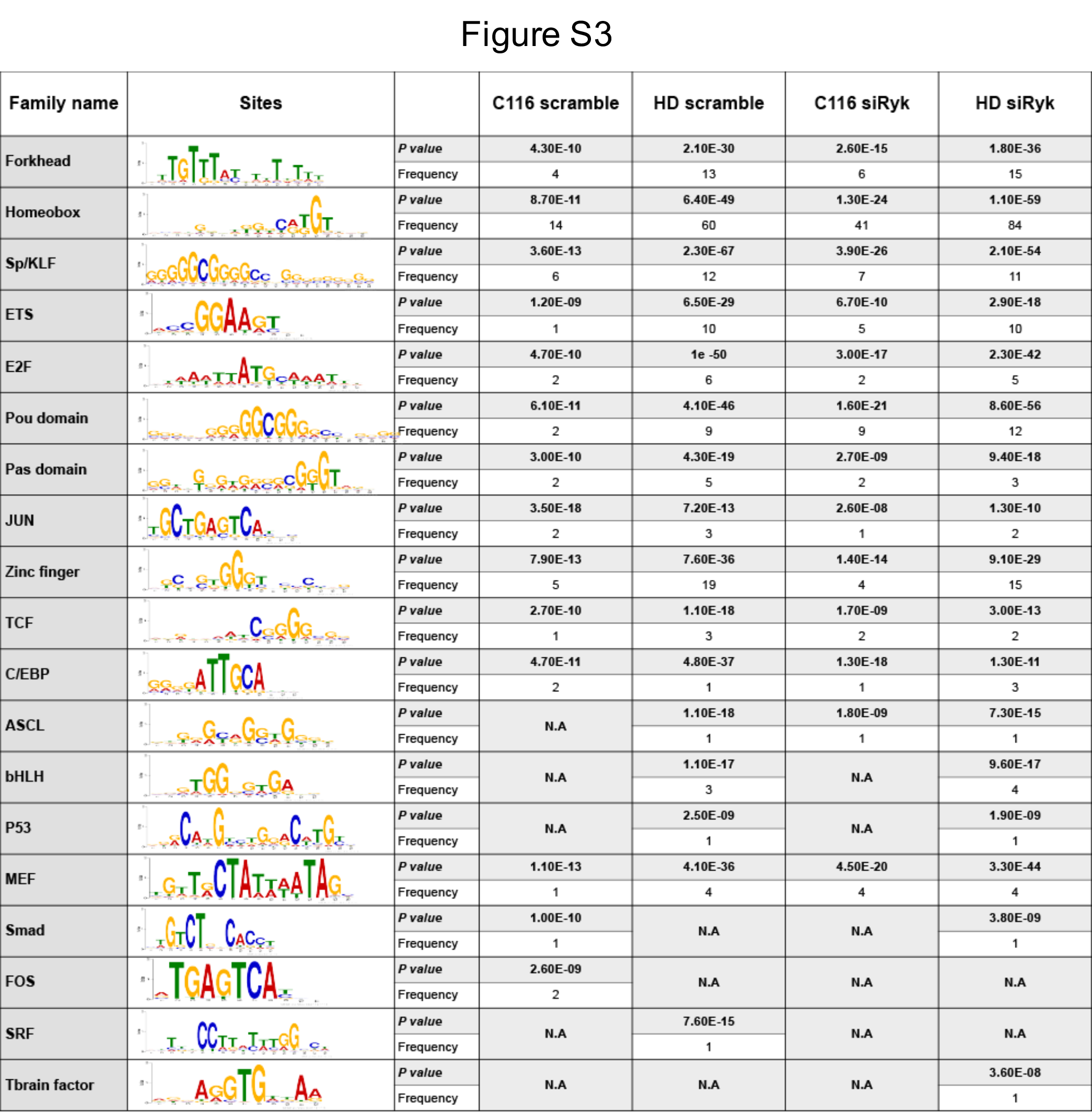
**

**FIGURE S3.** FOXO3 binding sites are enriched for candidate co-regulator motifs that are shared across *HTT* genotypes or unmasked in cells expressing mutant *HTT*.

**FIGURE S4.** Overlap between FOXO3 binding sites in human C116 neural stem cells and mouse neural stem cells. Overlap is shown for FOXO3 binding sites in mouse NSCs compared to a previously reported and comparable study of FOXO3 binding sites in mouse NSCs (Webb et al., 2013), using best human orthologs of mouse genes.

**FIGURE S5.** Network of FOXO3 direct targets in human HD NSCs compared to control cells. To maximize the assessment of biological relevance, FOXO3 direct targets defined by FOXO3 nuclear induction (F3T-IN, see Table S1/sheet 1) were used as seed genes to generate networks that include 200 high confidence neighbors as defined by a probability score ≥ 0.7 and edge information based on databases and experiments as available in the STRING database v10.0 (http://string-db.org/). This network is annotated with information on connectivity between F3T-INs with different regulatory profiles (gained or lost, positively or negatively regulated), strength of FOXO3 regulation upon FOXO3 nuclear induction, *bona fide* information from FOXO3 knockdown (F3T-IN-KD; see Table S1/sheet 2) and gene expression levels in HD NSCs versus corrected C116 NSCs as previously reported (Ring et al., 2015). Shown are networks of F3T that are conserved between normal and HD NSCs (*A*), reprogrammed (lost or gained) in a Ryk-independent manner in HD NSCs, regardless of their dependence on Ryk in normal *HTT* NSCs (*B*), and reprogrammed (lost or gained) in a Ryk-dependent manner in HD NSCs (*C*).

**FIGURE S6.** Gene target expression levels upon treatment with siRNAs and *HTT* expression levels upon reduction of *FOXO3* or reduction of *p16^INK4a^*. Data are mean±SD. (A) *FOXO3* mRNA levels are decreased by siRNA treatment HD and normal *HTT* NSCs. ****P* < 0.001 compared to non-targeting control (NTC) siRNAs. Related to Figure S6 and Figure 3A. (B) *ETS2* mRNA levels are decreased by siRNA treatment HD and normal *HTT* NSCs. ****P* < 0.001 compared to NTC siRNAs. Related to Figure 3B. (C) *FOXO3* mRNA levels upon overexpression of FOXO3-TM or FOXO3 in normal *HTT* or HD NSCs. ****P* < 0.001 and ***P* < 0.01 compared to empty vector. (D) Overexpressing FOXO3 or FOXO3-TM does not reduce the viability of human NSCs. (E) *ETS1* mRNA levels are decreased by siRNA treatment HD and normal *HTT* NSCs. **P* < 0.05 compared to NTC siRNAs. Related to Figure 3C. (F) *FOXO3* mRNA levels are decreased by siRNA treatment HD and normal *HTT* NSCs. ***P* < 0.01 and ****P* < 0.001 compared to NTC siRNAs. Related to Figure 6. (G) *p16^INK4a^* mRNA levels are decreased by siRNA treatment HD and normal *HTT* NSCs. ****P* < 0.001 compared to non-specific control siRNA treatment. Related to Figure 6. (H) *HTT* mRNA levels are unchanged by FOXO3 or p16^INK4a^ siRNA treatment in HD NSCs. ns, not significant.

**FIGURE S7.** Evaluation of candidate FOXO3 targets (*CDKN2AIP*, *SERTAD1*) and products of the *CDKN2A* locus (*i.e.*, p14^ARF^). Related to Figure 3. (A) *CDKN2AIP* mRNA levels are decreased by *FOXO3* silencing in stressed C116 NSCs, with no change detected in HD NSCs (left panel). *CDKN2AIP* mRNA levels are increased in HD NSCs (middle panel: ***P* < 0.01 compared to C116 cells). Growth factor (GF) deprivation increases *CDKN2AIP* mRNA levels in C116 NSCs (**P* < 0.05 compared to C116 cells) with no change detected in HD NSCs (right panel). ns, not significant. (B) *SERTAD1* mRNA levels are not altered by *FOXO3* silencing in HD and C116 NSCs (left panel). *SERTAD1* mRNA levels are slightly decreased in HD NSCs (middle panel: **P* < 0.05 compared to C116 cells). GF deprivation does not change *SERTAD1* mRNA levels in both cell genotypes (right panel). ns, not significant. (C) *p14^ARF^* mRNA levels are not altered by *ETS2* reduction in HD and C116 NSCs (left panel). *p14^ARF^* mRNA levels are increased in HD NSCs (middle panel: ****P* < 0.001 compared to C116 cells). GF deprivation does not change *p14^ARF^* mRNA levels in both cell genotypes (right panel). ns, not significant.

**FIGURE S8.** Increased levels of p16^INK4a^ and elevated SA-ß-gal activity are also characteristic of other non-isogenic HD NSC lines. Related to Figure 4. (*A*) p16^INK4a^ mRNA levels as determined by RT-PCR analysis in control NSCs (blue bars; MN08i-33114.B and ND42241) and HD NSCs (red bars; ND41656 - CAG 57 and ND42222 - CAG 109). Data are mean ± SD (N = 3). ****P* < 0.001 (one-way ANOVA; Tukey’s multiple comparison test). (B) Immunofluorescence analysis reveals robust increase of p16^INK4a^ in HD NSCs. Scale bar in all panels: 100 mm. (C) Representative images showing increased expression of SA-b-gal activity in HD NSCs (ND41656 - CAG 57; ND42222 - CAG 109) compared to control NSCs (MN08i-33114.B and ND42241). 10X magnification. Scale bar in all panels: 200 mm.

**FIGURE S9.** Relevant markers of senescence evaluated in HD NSCs and MSNs. Related to Figure 4 and Figure 5. (A) *p21^CIP1^* mRNA levels are decreased in HD compared to C116 NSCs (***P* < 0.01). (B) *p27^KIP1^* mRNA levels are unchanged in HD compared to C116 NSCs. ns, not significant. (C) *MMP-3* mRNA levels are increased in HD compared to C116 NSCs (****P* < 0.001). (D) Immunofluorescence analysis reveals depletion of HMGB1 from nuclei of HD MSNs compared to C116 MSNs. Scale Bar in all panels: 100 µm. (E) Quantification of nuclear HMGB1 pixel intensity for N = 437 C116 NSCs and N = 491 HD NSCs. Data are mean±SD (*****P* < 0.0001). (F) Quantification of nuclear area in HD (N = 1879 cells) and C116 (N = 2607 cells) MSNs. t-test **P* < 0.05. Data are mean±SEM.​

**
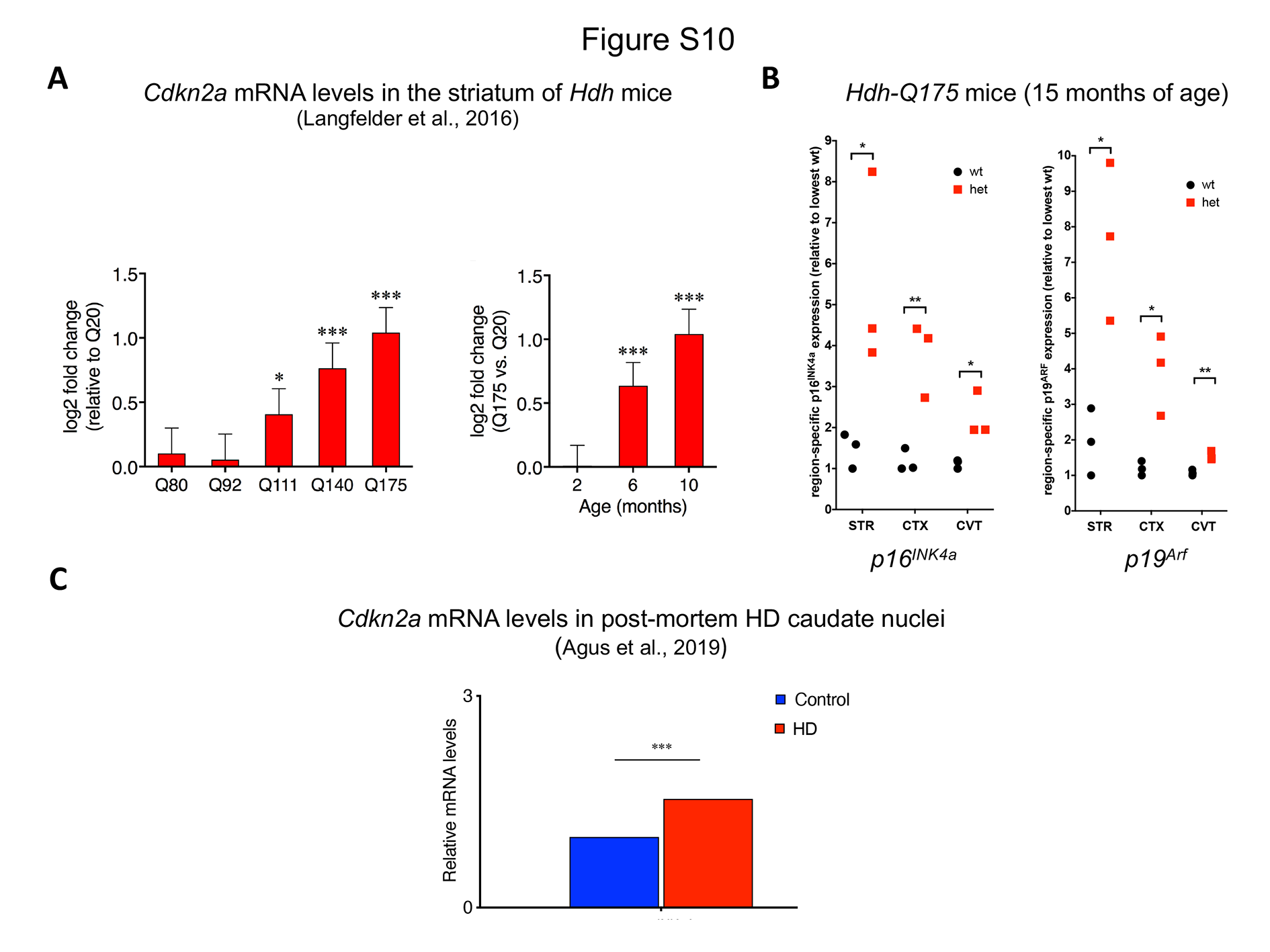
**

**FIGURE S10.** Increase of *p16^INK4a^* mRNA levels in the striatum of HD model mice. (A) CAG-repeat-length- and age-dependent increase of *Cdkn2a* mRNA levels. Graph representation of data by Langfelder and coll. (Langfelder et al., 2016). (B) *p16^INK4a^* mRNA levels are increased in the striatum (STR) of heteroztygote HD knock-in mice zQ175DN (het) compared to wild-type (wt) mice at 15 months of age, with lesser increases detected in the cortex (CTX) and in the cerebellum (CVT). Similar features were detected for *p19^Arf^* mRNA levels. t-test **P* < 0.01 and ***P* < 0.001. Data are expression calculated as relative to the *Hprt* housekeeping gene and then ratioed over the lowest wild-type value. (C) Increase of *CDKN2A* mRNA levels in *post-mortem* prodromal HD caudate nuclei. Graph representation of data by Agus and coll. (Agus et al., 2019). ****P* < 0.001.
